# Time course of western diet (WD) induced nonalcoholic steatohepatitis (NASH) in female and male *Ldlr*^*-/*^^*-*^ mice

**DOI:** 10.1371/journal.pone.0292432

**Published:** 2023-10-11

**Authors:** Melinda H. Spooner, Manuel Garcia-Jaramillo, K. Denise Apperson, Christiane V. Löhr, Donald B. Jump

**Affiliations:** 1 Nutrition Program, College of Health, Oregon State University, Corvallis, OR, United States of America; 2 Environmental and Molecular Toxicology, Oregon State University, Corvallis OR, United States of America; 3 Department of Biomedical Sciences, Carlson College of Veterinary Medicine, Oregon State University, Corvallis, OR, United States of America; Tokyo University of Agriculture, JAPAN

## Abstract

**Background:**

Nonalcoholic fatty liver disease (NAFLD) is a global health problem. Identification of factors contributing to the onset and progression of NAFLD have the potential to direct novel strategies to combat NAFLD.

**Methods:**

We examined the time course of western diet (WD)-induced NAFLD and its progression to nonalcoholic steatohepatitis (NASH) in age-matched female and male *Ldlr*^*-/-*^ mice, with time-points at 1, 4, 8, 20 and 40 weeks on the WD. Controls included *Ldlr*^*-/-*^ mice maintained on a purified low-fat diet (LFD) for 1 and 40 weeks. The approach included quantitation of anthropometric, plasma and liver markers of disease, plus hepatic histology, lipids, oxylipins, gene expression and selected metabolites.

**Results:**

One week of feeding the WD caused a significant reduction in hepatic essential fatty acids (EFAs: 18:2, ω6, 18:3, ω3) which preceded the decline in many C_20-22_ ω3 and ω6 polyunsaturated fatty acids (PUFA) and PUFA-derived oxylipins after 4 weeks on the WD. In addition, expression of hepatic inflammation markers (*CD40*, *CD44*, *Mcp1*, *Nlrp3*, *TLR2*, *TLR4*, *Trem2*) increased significantly in both female & male mice after one week on the WD. These markers continued to increase over the 40-week WD feeding study. WD effects on hepatic EFA and inflammation preceded all significant WD-induced changes in body weight, insulin resistance (HOMA-IR), oxidative stress status (GSH/GSSG ratio) and histological and gene expression markers of macrosteatosis, extracellular matrix remodeling and fibrosis.

**Conclusions:**

Our findings establish that feeding *Ldlr*^*-/-*^ mice the WD rapidly lowered hepatic EFAs and induced key inflammatory markers linked to NASH. Since EFAs have an established role in inflammation and hepatic inflammation plays a major role in NASH, we suggest that early clinical assessment of EFA status and correcting EFA deficiencies may be useful in reducing NASH severity.

## Introduction

Nonalcoholic fatty liver disease (NAFLD) is the most common form of chronic fatty liver disease worldwide. Approximately 25% of the global population is estimated to have some level of NAFLD [1–3]. The World Health Organization in 2016 reported over 1.9 billion overweight adults, which paralleled the increase in patients diagnosed with NAFLD [1,2,4,5]. The National Health and Nutrition Examination Survey (NHANES) estimated nearly 40% of adults in the US are obese [2]. Obesity severity increases the likelihood of developing NAFLD ranging from 75% in overweight individuals to 90–95% in morbidly obese individuals [1,2,6,7]. NAFLD is associated with metabolic syndrome (MetS) and MetS is linked to obesity, type 2 diabetes mellitus (T2DM), dyslipidemia and hypertension, the top four risk factors for NAFLD [1,4].

NAFLD is a continuum of fatty liver diseases ranging from hepatic macrosteatosis to nonalcoholic steatohepatitis (NASH, the progressive form of disease), cirrhosis, hepatocellular carcinoma (HCC) and liver failure [8,9]. NAFLD occurs in children and adults, and both males and females [1]. Factors contributing to the onset and progression of NAFLD include diet, lifestyle, genetics, sex, ethnicity and genetic polymorphisms. Since there are no FDA-approved treatment strategies for NAFLD, current treatment strategies focus on treating the comorbidities associated with NAFLD, such as obesity, type 2 diabetes, insulin resistance and dyslipidemia [1,4,10].

According to the National Institute of Diabetes and Digestive and Kidney Disease (NIDDK), “NAFLD is a silent disease with no obvious symptoms” https://www.niddk.nih.gov/health-information/liver-disease/nafld-nash/symptoms-causes. As such, NAFLD is typically diagnosed in patients with pre-existing diseases, such as obesity, type 2 diabetes, dyslipidemia and metabolic syndrome (MetS). The current view of NAFLD onset and progression to nonalcoholic steatohepatitis (NASH) involves the accumulation of hepatic lipid (steatosis) including excessive hepatic cholesterol, diacylglycerols, ceramides, and oxidized lipids that promote inflammation, hepatic injury and fibrosis [11–14]. In addition, the gut-liver axis plays a role in the onset and progression of NAFLD [15–18]. The liver is the first organ to encounter dietary products absorbed by the gut [19]. Problems occur when the integrity of the gut is compromised, such as leaky gut that can lead to gut luminal products entering the bloodstream. Some of these products are derived from gut microbes and are known to promote systemic inflammation. For example, NAFLD is associated with a low-grade metabolic endotoxemia [20,21]. Endotoxin (lipopolysaccharide, LPS) is a gram-negative bacterial cell wall component that activates the innate immune system through Toll-like receptors, specifically TLR4. This process likely contributes to hepatic inflammation and dysregulation of hepatic metabolism. Interestingly, germ-free mice do not develop diet-induced fatty liver disease [22] revealing the importance of diet and gut-microbes in the onset and progression of NAFLD.

The diet most commonly associated with NAFLD is the western diet (WD) [23–29]. The WD is high in saturated (SFA) and monounsaturated fat (MUFA), cholesterol, simple sugar, and low in fiber. The WD also has an abnormal content of essential fatty acids (EFAs), i.e., linoleic acid (LA, 18:2, ω6) and α-linolenic acid (ALA, 18:3, ω3). The EFAs are either below recommended intake levels or the WD has a high ω6/ω3 polyunsaturated fatty acid (PUFA) ratio [4]. A high ω6/ω3 PUFA ratio is associated with inflammation [30]. PUFA also represent a low percentage (less than10%) of all fatty acids in the WD. Several clinical reports indicate that NAFLD severity, i.e., progression from steatosis to NASH, cirrhosis and HCC, is associated with a decline in hepatic C_18-22_ ω3 and ω6 PUFA [31–39]. Preclinical studies have established that the WD-induced decline in hepatic C_18-22_ ω3 and ω6 PUFA is associated with NAFLD onset and progression to NASH [40–46].

While clinical and histological markers of NAFLD are well-defined, the role of chronic ingestion of a WD-like diet on the onset of NAFLD and its progression to NASH has not been fully elucidated. There is little data in the human or preclinical literature on the antecedent events preceding the abnormal accumulation of lipids in the liver. Our aim in this report is to fill this gap by using an established preclinical model of WD-induced NAFLD [42–45,47] to identify early changes in systemic and hepatic markers that precede overt evidence of insulin resistance, obesity and NAFLD. Accordingly, we fed age-matched female and male *Ldlr*^*-/-*^ mice a purified low-fat diet (LFD) and a western diet (WD) for 40 wks to promote NASH. Our use of the *Ldlr*^*-/-*^ mouse as a preclinical model of NAFLD is based on the following information. *Ldlr*^*-/-*^ mice used in this study are on the C57BL/6J background. In contrast to wild type C57BL/6J mice fed the WD, *Ldlr*^*-/-*^ develop the full spectrum of NASH characteristics in response to the WD [43–45,47,48]. Interestingly, recent studies indicate that excessive hepatic cholesterol content plays a significant role in human NAFLD and NASH [49–53]. Moreover, cholesterol lowering drugs, like statins and/or ezetimibe, are used in ~52% of diabetes patients to improve dyslipidemia and in NAFLD patients to reduce the risk of HCC [52,54].

WD-fed *Ldlr*^*-/-*^ mice become obese and develop multiple markers of NAFLD including insulin resistance (HOMA-IR), dyslipidemia, endotoxemia and elevated markers of hepatic macrosteatosis, inflammation, fibrosis as well as evidence of hepatic injury [alanine aminotransferase (ALT) and aspartate aminotransferase (AST)]. In addition, the WD has major effects on the hepatic lipidome, including changes in hepatic oxylipins [43–45]. Since previous studies documented an inverse relationship between NAFLD severity and the hepatic oxylipidome [43,47], we were interested in determining whether WD rapidly alters the hepatic oxylipidome. Finding rapid effects on the hepatic PUFA and the oxylipidome may reveal a potential cause and effect role for specific PUFA and oxylipins in the onset and progression of NAFLD and NASH.

Our goals in this study were to: 1) identify early markers of WD-induced disease; 2) determine which lipids might serve as biomarkers of NAFLD to NASH; and 3) determine if male and female mice respond similarly to the WD. Herein, we establish that one week on the WD was sufficient to induce significant changes in multiple systemic and hepatic markers linked to NASH, including dyslipidemia and inflammation, as well as changes in hepatic essential fatty acid (EFA) content. These early responses to the WD likely set the stage for disease progression resulting in significant liver injury and NASH.

## Materials and methods

### Animals and diets

All procedures for the use and care of animals used in laboratory research were followed and approved by the Institutional Animal Care and Use Committee at Oregon State University (OSU). The study used two-month-old female and male *Ldlr* ^*-/-*^ [B6;129S7- *Ldlr* ^*Tm1Her*^/J mice, stock# 002207] purchased from Jackson Laboratories. The study was carried out concurrently with both female and male mice. Upon arrival, mice were housed (5 mice/cage) at the OSU Linus Pauling Science Center vivarium in the same temperature-controlled room and handled by the same personnel throughout the study. Mice were maintained on a 12-hour light/dark cycle.

**Fig 1:**
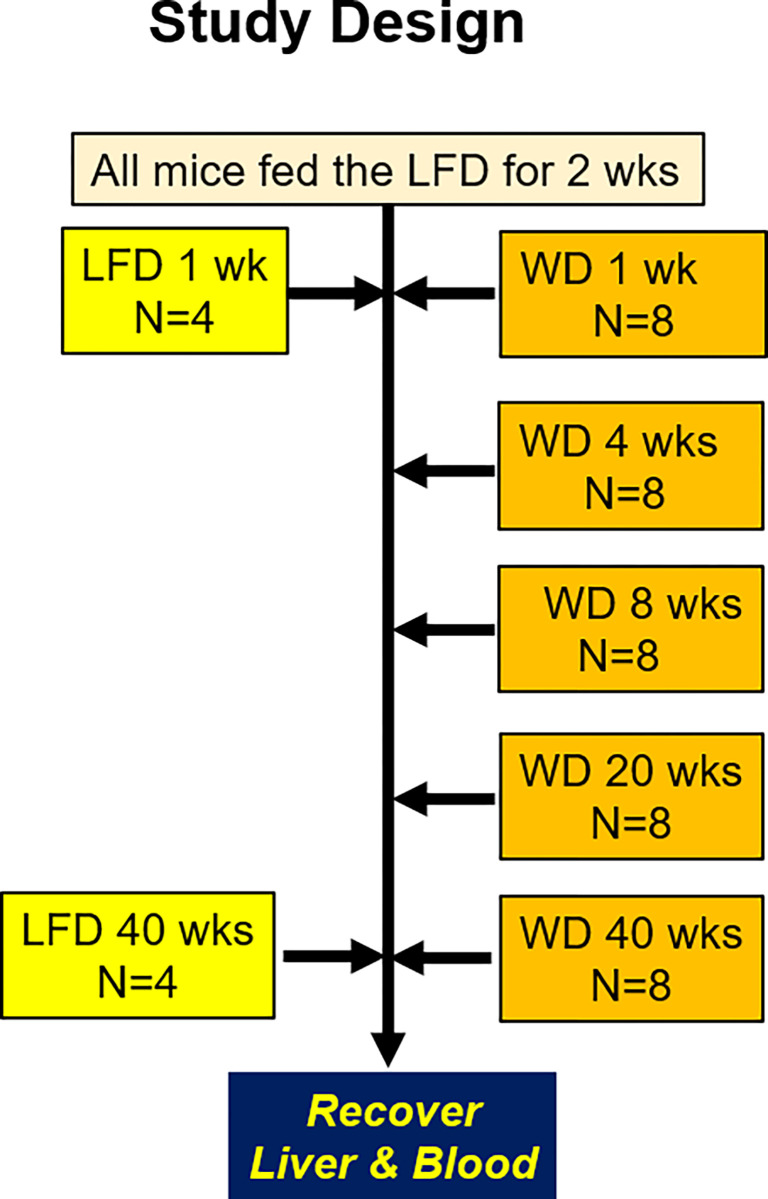
Study design for the assessment of western diet (WD) effects on female and male *Ldlr*^*-/-*^ mice. Mice were fed a low-fat diet (LFD) and a western diet (WD) for 1 to 40 weeks (wks). The diagram illustrates the time course and the number of female and male mice used in the study. The study was carried out concurrently with age-matched female and male mice. The study used a total of 48 female and 48 male *Ldlr*^*-/-*^ mice.

The study design is illustrated in **Fig 1**. All mice were fed a purified low-fat diet (LFD) [Research Diets: D12450K] for 2 weeks (wks) prior to initiating the feeding trial to acclimate the mice to a purified diet and the vivarium. The 40-week (wk) time course study of female and male mice consisted of two randomized groups for each sex. The Low-Fat Diet (LFD) [Research Diets: D12450K] group and the Western Diet (WD) [Research Diets: D12079B] group. The purified low-fat diet (LFD) contained 20% energy as protein (casein, cysteine), 70% energy as carbohydrate (corn starch (52%); maltodextrin (14%); sucrose 0.4%)); 10% energy as fat (soybean oil, lard) and cholesterol (0.002 mg/g) of diet. The purified western diet (WD) contained 17% energy as protein (casein, methionine); 43% energy as carbohydrate (sucrose (30%); corn starch (10%); maltodextrin (3%)); 40% energy as fat (butter, corn oil); and cholesterol at 1.5 mg/g of diet. Both diets contained a vitamin and mineral mix and fiber, while the WD contained an additional antioxidant. The energy density of the LFD and WD was 3.82 kcal/gram and 4.67 kcal/gram, respectively. More details of the diets, including the fatty acid composition of the diets, are described in **S1A Fig**.

Female and male mice were maintained on the LFD for 1 and 40 wks (4 mice/sex and time point). The remaining female and male mice were fed the WD (8 mice/sex and time point). Health status and body weight of the mice were assessed twice weekly. Chow remaining from the previous feeding was weighed, discarded and fresh food added.

### Euthanasia: Recovery of blood and liver

After 1 and 40 wks on the LFD and 1, 4, 8, 20 and 40 wks on the WD, mice were fasted overnight. The next morning at 8 AM, mice were euthanatized by CO_2_ and liver and blood were collected as described above [43,44]. A portion of the liver (major lobe) was transferred to vials containing PBS-buffered formalin for histology. The remaining liver was frozen in liquid nitrogen and subsequently transferred to a -80°C. freezer for storage. Collected blood was transferred to vials containing EDTA to prevent clotting. Plasma and cells were centrifuged to recover cells and plasma separately and stored at -80°C. Liver and plasma were analyzed as described in the Methods section above.

### Plasma and hepatic assessments

Plasma glucose, total and free cholesterol (T Chol; F Chol), and triacylglycerides (TAG) were measured using kits (Wako Diagnostics; Richmond, VA) as previously described [41]. Plasma insulin (Crystal Chem; sales@crystalchem.com), aspartate aminotransferase (ALT), and alanine aminotransferase (AST) were measured using kits from Thermo-Fisher as described [41]. Plasma non-esterified fatty acids (NEFA) were measured using a kit from Sigma-Aldrich and β-hydroxybutyrate (β-HB) was measured with a kit from Zen-Bio. Plasma toll-like receptor agonists (TLR2 Ag and TLR4 Ag) were quantified as previously described [46]. The homeostatic model of insulin resistance (Homa-IR) was calculated as described previously [55].

### Liver histology

After euthanasia of the mice, approximately 100 mg of fresh mouse liver from each animal was fixed in buffered formalin, paraffin embedded, sliced and stained with hematoxylin-eosin (H & E) or Picro Sirius red (PCR) (Nationwide Histology, Veradale, WA) [43,44]. Each slide contained 2 to 4 liver slices. Histological analysis and scoring for microsteatosis and macrosteatosis, inflammation (infiltrated leukocytes) and fibrosis were provided by two investigators: Christiane V. Löhr, Dr. Med. Vet., PhD, board-certified by the American College of Veterinary Pathologists; and K. Denise Apperson, DVM, PhD) using a modified Kleiner scoring system established for mouse models of NAFLD as described previously [43,56]. Histological samples were blinded as to sex, timepoint and diet. Descriptive statistics were performed in Microsoft Excel for Mac 2011 (v 14.7.2; www.microsoft.com) and StatPlus for Mac (v6, www.analystsoft.com/en). Digital images were taken with a Nikon Eclipse 6 microscope and digital camera (Mpixel) and NIS-BR Elements imaging software (v21.1; www.nikonmetrology.com). Digital images taken at 400x magnification were used for steatosis scoring (see **S1B and S1C Fig**). An effort was made to place the central vein of a lobule at one of the corners of the image so that each image covered at least one-quarter of that lobule, including all three zones of the hepatic lobule.

Steatosis was objectively analyzed as the average percent surface area occupied by vacuoles using the image analysis software, ImageJ (https://imagej.nih.gov/ij/). Two images were taken of H&E-stained sections at 400x and the percent of affected surface area was calculated for each. The two values were then averaged. Steatosis was subjectively analyzed as the percent of affected surface area observed on H&E-stained slides at 100x (10x objective) and 400x magnifications. Vacuolization was characterized as macrovesicular, in which vacuoles displace hepatocyte nuclei, or microvesicular. Macrovesicular and microvesicular steatosis was scored separately. Severity was scored using the scale: 0 (0%), 1 (>5% but <33%), 2 (>33% but <66%), 3 (>66%). When possible, the distribution of vacuoles was described as pericentral, midzonal, or periportal.

Inflammation was defined as intralobular inflammatory foci of at least 5 leukocytes associated with disruption of hepatic plates or increased hepatocellular eosinophilia. Inflammation scores were calculated as the total number of clusters averaged over 5 fields in H&E-stained tissues examined at 100x (total number of clusters in 3.1 mm^2^) (**S1B Fig**). The following scale was used: normal 0 (<0.5 foci), slight 1 (<0.5–1 foci), moderate 2 (1–2 foci), severe 3 (>2 foci).

Fibrosis was also objectively quantified as percent surface area occupied by Sirius Red-stained collagen by image analysis using ImageJ. Two images were taken at 100X from the liver section of each mouse and the calculated percent areas were averaged. Fibrosis was subjectively analyzed to determine severity and distribution patterns. Distribution of fibrosis was described as perisinusoidal, periportal, pericentral, or bridging (**S1D Fig**). The following scale was used: absent (0), mild (1), moderate (2), or severe (3).

### Hepatic lipids and metabolites

Hepatic lipids were extracted and process for gas chromatography (GC), ultra-high-performance liquid chromatography coupled to tandem mass spectrometry (UPLC/MS/MS) and liquid chromatography coupled to tandem mass spectrometry (LC/MS/MS) as described [43,47]. Fatty acid standards for the GC analysis were purchased from Nu-Chek Prep and Cayman Chemical Company. Oxylipin standards were purchased from Cayman Chemical Company [47]. Hepatic glutathione (GSH and GSSG) and selected metabolites were fractionated and quantified using the metabolomic approach described previously [57], while hepatic bile acids were fractionated and quantified as described [58].

### RNA extraction and gene expression analysis

Liver RNA was extracted using Trizol (Life Technologies) [43,45,46], quantified and used for qRTPCR as described previously [43,47]. The selection of quantified transcripts for this report was based on our previous studies [43,47]. Cyclophilin was used on the reference gene and mRNA abundance was reported as mRNA abundance Delta C_T_. Results in heat maps are reported as mean Delta Ct values at each diet and time point. Results in the graphed data are reported as the mean ± SEM for the Delta Ct values for each diet and time point. **S1 Table** contains a list of all genes examined and the primers used for qRTPCR quantitation of mRNA abundance.

### Statistical analysis and data presentation

All data presented in this report was assessed for statistical significance using the statistical packages in MS-Excel and MetaboAnalyst 5.0 (http://www.metaboanalyst.ca/MetaboAnalyst/) [59–61]. The number of female and male mice in each group is displayed in **Fig 1**. ANOVA with Tukey’s HSD post-hoc test was used to identify significant differences (p-value < 0.05) between groups and the false discovery rate (FDR). The FDR and the results of Tukey’s HSD are reported in Supplementary Data: (**S2 Table** All Female data PLoS1 2023**; S3 Table** All Male data PLoS1 2023**)**. Volcano plots were prepared using MetaboAnalyst. Excel was used to calculate fold-change and T-test for the 1 and 40 wk comparisons between LFD- and WD-fed mice.

Data is presented in graphs, heat maps and a table. Graphs were prepared using MS-Excel and the data is represented as mean ± standard error of the mean (SEM). The heat maps were prepared using the following program (https://software.broadinstitute.org/morpheus/). The mean value for each item within each group (time and diet) was used to prepare the heat maps. Significant difference (p <0.05) at 1 and 40 wks between WD and LFD fed mice in the heat maps and graphed data is designated with an asterisk (_ӿ_).

## Results

### Food consumption and body weight

**Fig 2:**
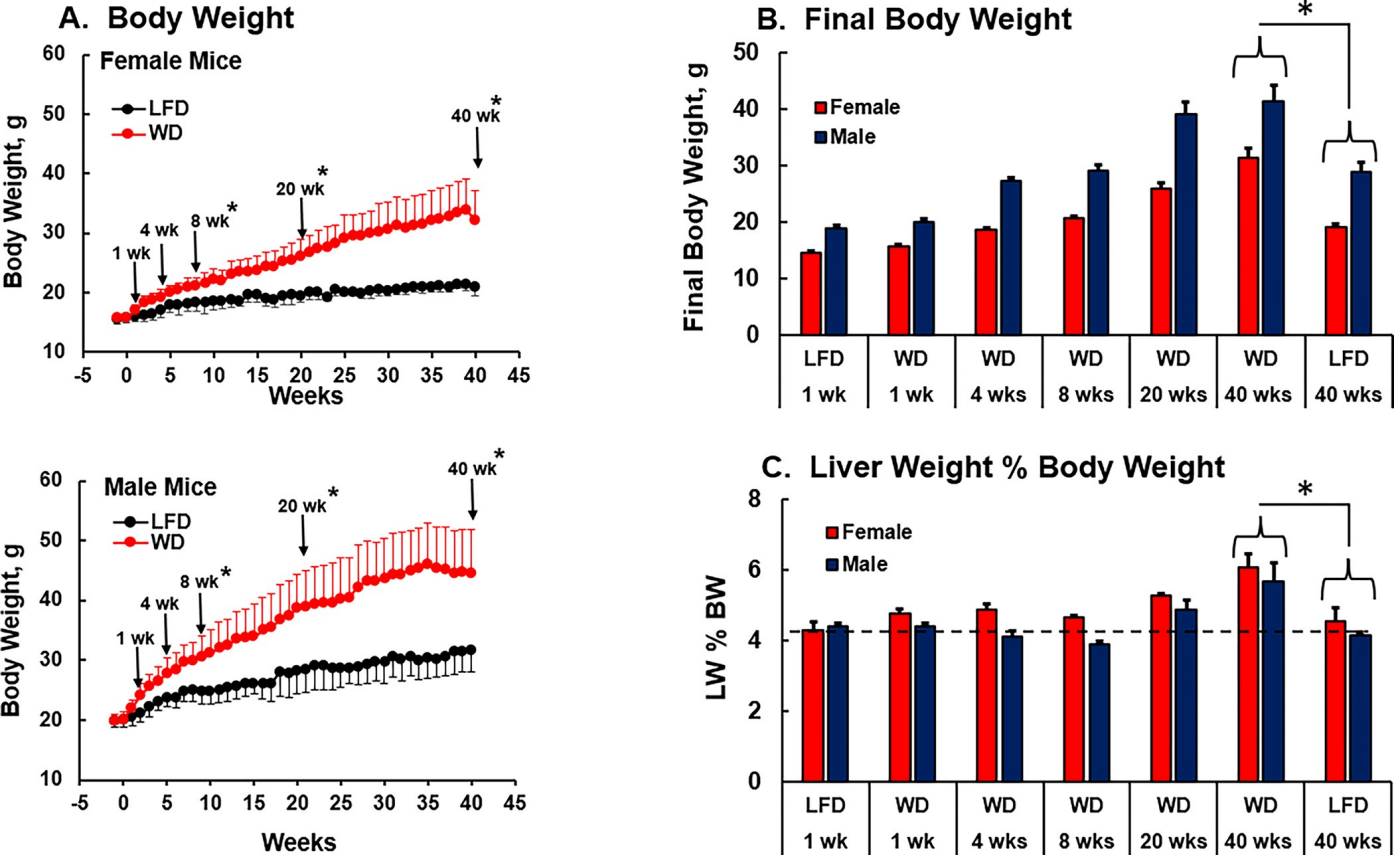
Diet effects on body and liver weight. [**A**] Body Weight: Body weight was measured 1 wk prior to starting the feeding trial while all mice were on the LFD and twice weekly after starting the feeding trial for all LFD and WD-fed mice in the colony for the 40 wk feeding study. Results are presented as average weight (g) ± SEM. After 1, 4, 8, 20 and 40 wks on the LFD and WD, mice were fasted overnight and euthanatized the next morning to recover blood and liver, as described in Methods. Final body weight and liver weight was obtained and results are reported as **[B]** Final body weight (mean ± SEM) and **[C]** Liver weight as a % of body weight: LW%BW, mean ± SEM. Significant differences (p<0.05) between WD and LFD fed mice are designated by an asterisk, (ӿ).

Female and male mice fed the LFD and WD consumed ~2.5 grams of the purified diet per mouse per day over the 40-week feeding period (**S1E Fig**). There was no evidence of WD-induced hyperphagia. The caloric density of the LFD and WD is 3.82 and 4.67 kcal/g, respectively. The cumulative food consumption, expressed as Kcal, over the 40 wk time course study is reported in **S1E Fig**. Mice fed the WD consumed more calories than mice fed the LFD leading to greater weight gain in WD-fed mice when compared to mice consuming the LFD (**Fig 2A**). Significant differences in body weight between mice fed the WD and LFD fed groups were achieved after 8 wks on the purified diets. Overall, female and male mice fed the WD gained 80.2% and 76.3% more weight than age-matched female and male mice fed the LFD for 40-wks, respectively.

Female and male mice were euthanized at the times indicated in **Fig 1** after an overnight fast (Methods). Diet effects on the final body weight and liver weight, reported as Liver weight % of Body Weight (LW%BW), are illustrated in **Fig 2B & 2C**. Body weight of both female and male mice increased over time on both diets with males gaining more weight than female mice. As noted in **Fig 2A**, statistical differences in body weight (WD versus LFD) were detected after 8 wks on the WD. In **Fig 2B**, statistical differences in body weight between age-matched LFD and WD fed mice are apparent after 40 wks.

We previously reported that mice fed the WD have increased liver weight when compared to mice fed the LFD [43,44]. Liver weight presented as a % of body weight (LW%BW) is illustrated in **Fig 2C**. Mice fed the LFD maintain LW%BW at ~ 4% throughout the feeding studies. The dashed line in **Fig 2C** provides a comparison of LW%BW across all groups. LW%BW increased significantly to ~6% in both female and male mice fed the WD for 40 wks. The earliest change in LW%BW in response to WD in both female and male mice was after 20 wks in both female and male mice fed the WD.

### Western diet effects on plasma markers

**Fig 3:**
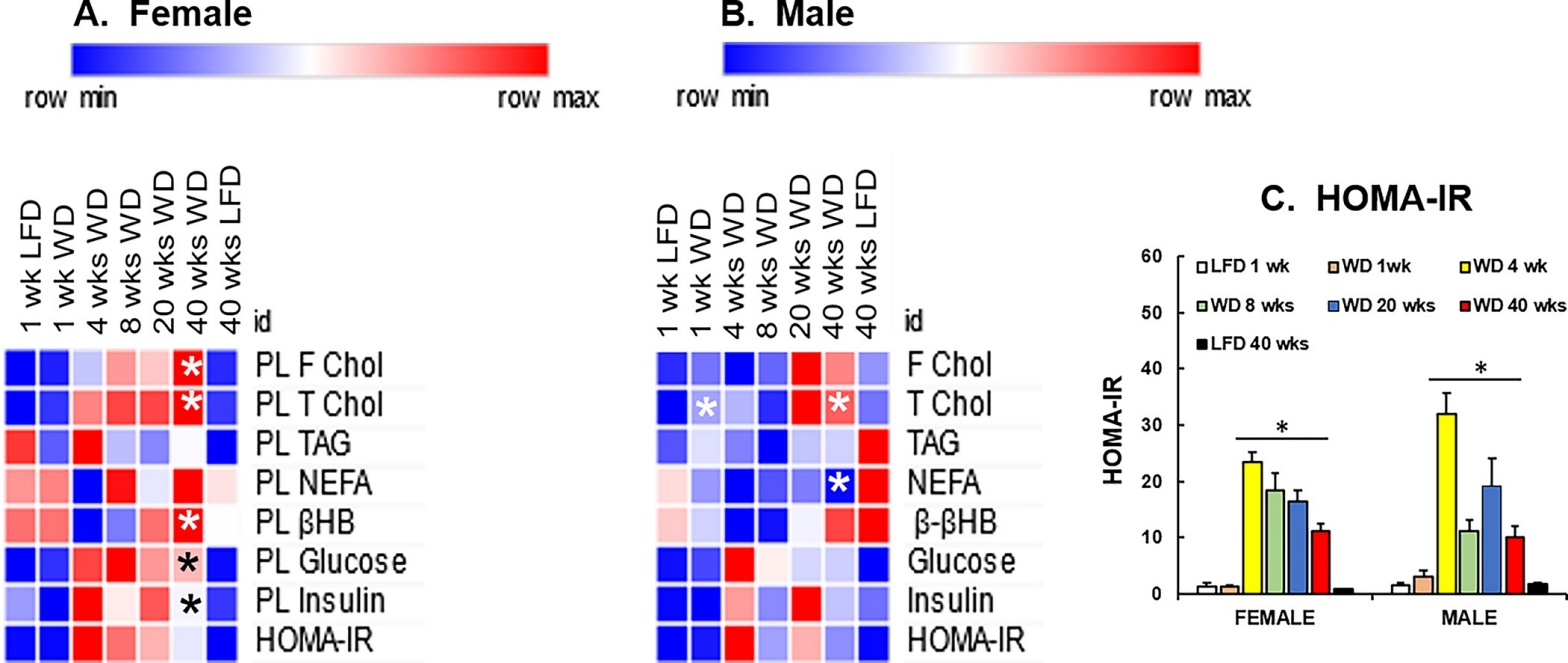
Time-course of WD effects on plasma lipids, glucose and insulin in female and male *Ldlr*^*-/-*^ mice. **A & B:** Plasma collected at euthanasia was used to quantify free cholesterol (F Chol), total cholesterol (T Chol), triacylglycerol (TAG), non-esterified fatty acids (NEFA); β-hydroxybutyrate, (βHB); glucose, insulin. The glucose and insulin data were used to calculate Homa-IR (Homeostatic Model Assessment of Insulin Resistance. Significant differences (p > 0.05) between the WD and LFD groups at 1 and 40 wks are shown by an asterisk (ӿ). **C:** Homa-IR is presented in graphic form. The statistical difference (p<0.05) between WD (wks 4–40 wks) and LFD-fed female and male mice (at 1 and 40 wks) is designated by an asterisk (ӿ).

WD effects on plasma markers associated with dyslipidemia [total and free cholesterol (T Chol, F Chol), triacylglycerides (TAG)], non-esterified fatty acids (NEFA), β-hydroxybutyrate, (βHB)] and glucose, insulin & insulin resistance (Homa IR) are presented in **Fig 3**. Plasma total cholesterol (T-Chol) and free cholesterol (F-Chol) in female mice trended upward after 4 wks on the WD and increased significantly over the 40 wks of WD feeding (**Fig 3A**). In male mice, T Chol was significantly increased after 1 wk and 40 wks on the WD, whereas F-Chol trended upward after 8 wks on the WD (**Fig 3B**). These results are expected since the WD is moderately high in cholesterol content (0.15% w/w, (**S1A Fig**) and *Ldlr*^*-/-*^ mice have impaired LDL-cholesterol clearance from blood [62]. While plasma TAG, NEFA and βHB changed significantly across the seven groups of mice based on the FDR values (see, **S2 and S3 Tables**) the pattern of change appeared random and did not follow a consistent pattern.

Plasma glucose, insulin and the measure of insulin resistance, i.e., homeostatic model assessment of insulin resistance (Homa-IR) (**Fig 3C**) changed significantly over the 40 wks of WD feeding. These values were unaffected by the WD after 1 wk, but increased significantly after 4 wks on the WD and remained elevated throughout the WD feeding period, when compared to LFD-fed mice. These results indicate that the WD promotes dyslipidemia within 1 to 4 wks on the WD and insulin resistance within 4 wks on the WD in both female and male mice.

**Fig 4:**
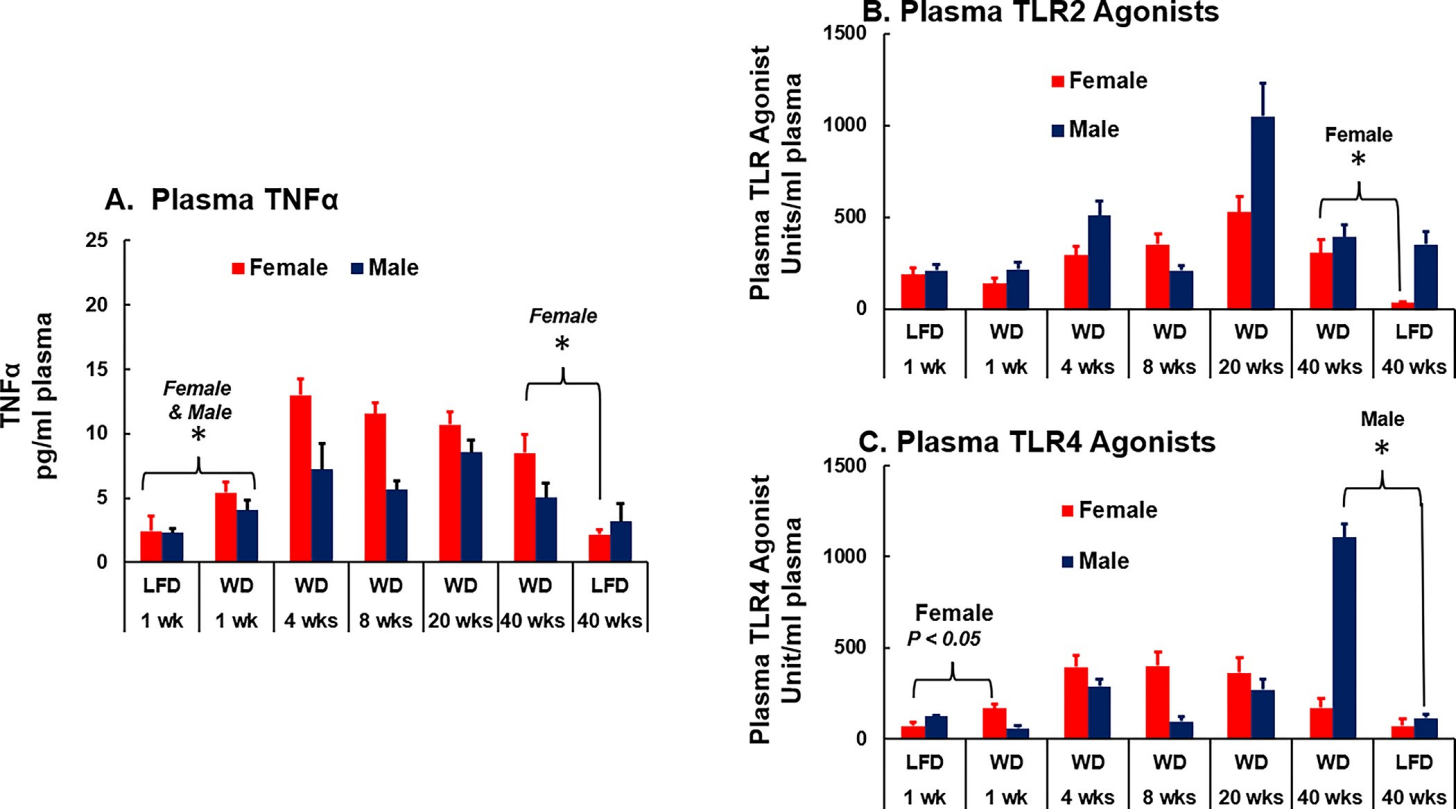
Time-course for LFD and WD effects on plasma markers of inflammation. Plasma from LFD and WD fed *Ldlr*^*-/-*^ female and male mice was collected and assayed for TNFα [A] and TRL2 [B] and TLR4 agonists (TLR-Ag) [C]. Results are presented as: TNFα, pg/ml plasma; TLR-Ag, units/ml plasma. Significant differences (p > 0.05) between the WD verses LFD groups at 1 and 40 wks are designated by an asterisk (ӿ).

The WD also affects plasma markers of inflammation (**Fig 4A–4C)**. Tumor necrosis factor (Tnfα) was significantly higher after 1 wk on the WD in both female and male mice (**Fig 4A**). After 40 wks on the WD Tnfα levels were significantly higher in female, but not male mice when compared to LFD-fed mice.

We also measured plasma Toll-like receptor agonists (TLR-Ag) (**Fig 4B & 4C**). TLR-Ag are pathogen associated molecular patterns (PAMPs) and likely represent microbial products appearing in plasma. For example, we previous reported the presence of endotoxin, a TLR4 agonist, in plasma of WD-fed mice [40]. Plasma TLR2-Ag trended upward after 4 wks on the WD in female and male mice (**Fig 4B**). After 40 wks on the WD, TLR2-Ag were significantly higher (~10-fold) in female, but not male mice. TLR4-Ag were significantly higher (~3-fold) in female mice after 1 wk on the WD (**Fig 4C**). While TLR4-Ag trended upward after 4 to 8 wks on the WD in female mice, plasma levels of these agonists decreased after 40 wks on the WD. The WD had little effect on plasma TLR4-Ag prior to 20 wks on the WD in male mice. After 20 and 40 wks on the WD plasma TLR4-Ag increased 2.4- and 9.8-fold, respectively. These results reveal a different timeline for the WD to alter plasma levels of TLR2-Ag and TLR4-Ag in female and male mice. While the WD effects on plasma TNFα are rapid, increasing after 1 wk on the WD, WD effects on plasma TLR2-Ag and TLR4-Ag require 4 wks on the WD to show an increase in plasma levels in female and male mice.

### WD effects on hepatic steatosis

**Fig 5:**
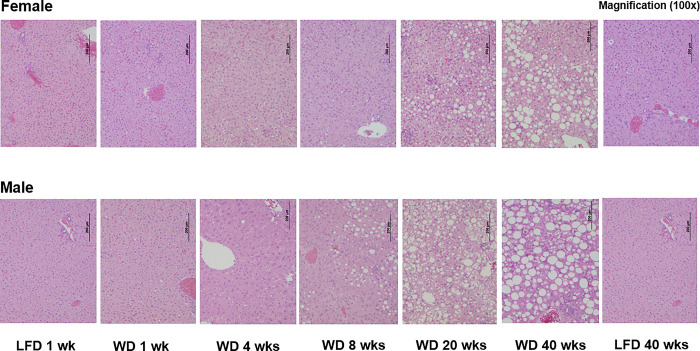
Time course of WD induced hepatosteatosis. Representative histologic images of hematoxylin-eosin (H & E)-stained liver from LFD and WD fed female and male *Ldlr*^*-/-*^ mice. Magnification: 100X. Histological scoring for microsteatosis (MIS) and macrosteatosis (MAS) is reported in **Fig 6**.

Histology is the gold standard for clinical assessment of NAFLD status. Hepatic histology was scored by two veterinary pathologists (**see** **Methods and Figs 5 & S1**). Both microsteatosis (**MIS**) and macrosteatosis (**MAS**) were scored. MAS is readily apparent at 100X magnification, while visualization of MIS required higher magnification (400x). MAS is defined as large lipid droplets in hepatocytes displacing nuclei, whereas the MIS score represents small lipid droplets that do not displace nuclei. Representative images of MIS and MAS are shown in the Supplement (**S1 Fig**).

**Fig 6:**
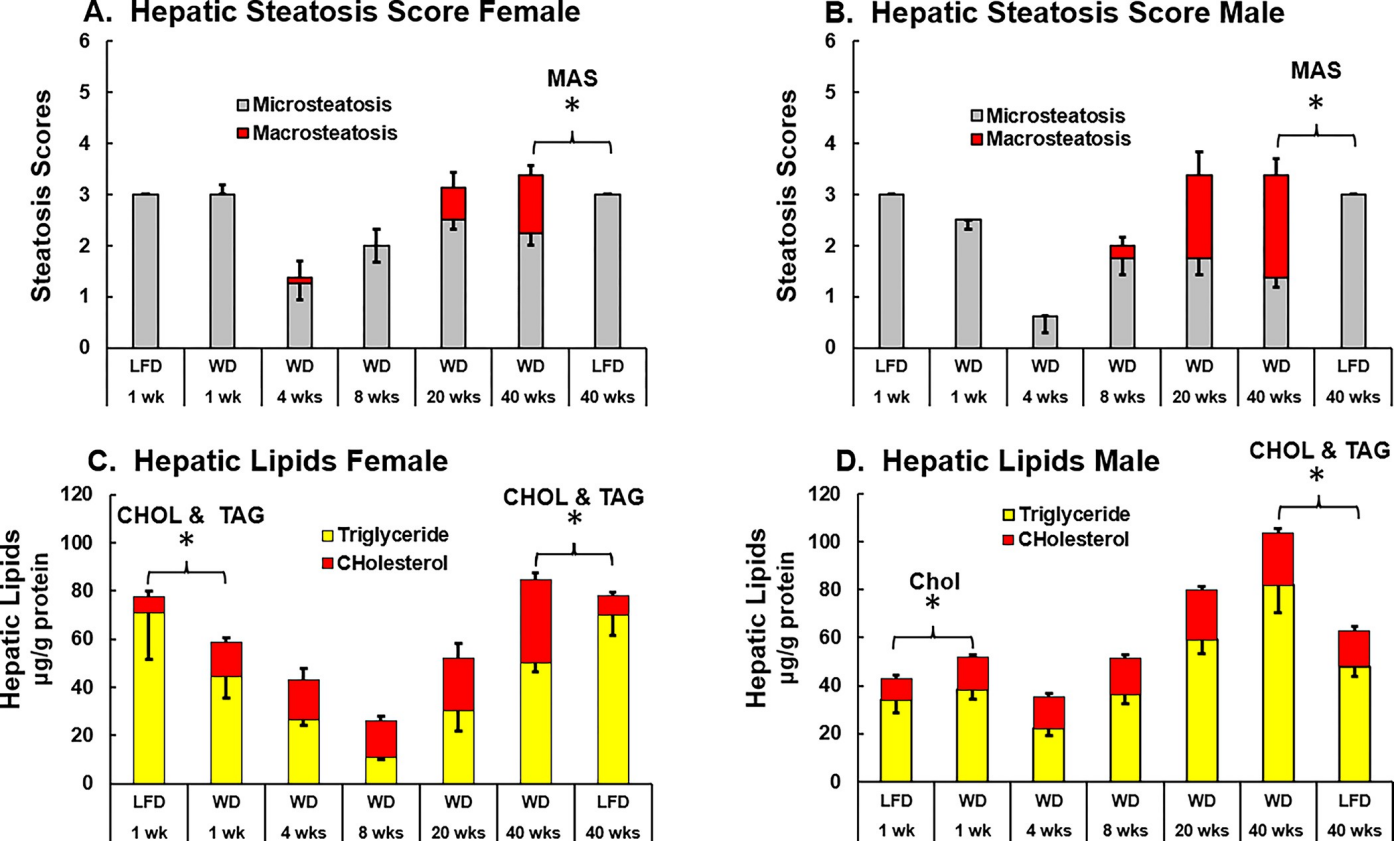
Histological scoring for hepatosteatosis and hepatic cholesterol and triglyceride content. Microsteatosis (MIS) and macrosteatosis (MAS) were scored on a 0 to 3-point scale for female **[A]** and male **[B]** mice. Hepatic triglyceride (TAG) and total cholesterol (CHOL) content are reported as μg/g protein in female **[C]** and male **[D]** mice. All scoring and direct measures of lipids are presented as the mean ± SEM. Significant differences (p<0.05) between WD versus LFD groups at 1 and 40 wks are designated by an asterisk (ӿ).

MAS was not detected in livers of female and male mice fed the LFD for 1 or 40 wks, or in mice fed the WD for 1 to 4 wks (**Fig 5**). MAS, however, was observed in male and female mice as early as 8 and 20 wks on the WD, respectively. Scoring for MIS revealed changes over the 40 wk WD feeding trial in both female and male mice. Briefly, MIS decreased in livers of WD-fed mice at 4 wks and increased afterward (**Fig 6A & 6B**). Whereas MAS increased after 8 wks on the WD in both female and male mice.

Since lipid droplets (steatosis) contain neutral lipids, both triglycerides (TAG), cholesterol (CHOL) esters, we quantified hepatic TAG and CHOL in LFD- and WD-fed female and male mice **(Fig 6C & 6D).** While the relative abundance of TAG and CHOL remained relatively unchanged after 40-wk on the LFD, hepatic TAG and CHOL changed significantly over 40 wks of feeding the WD in both female and male mice. In female mice, there was a progressive decrease in TAG, but not CHOL from 1 to 8 wks on the WD; afterward both lipid classes increased in liver. Total CHOL increased ~2-fold after 1 wk on the WD and this amount of CHOL does not change until 20 and 40 wks of WD feeding where CHOL increased ~5-fold when compared hepatic CHOL in 40 wk LFD-fed mice. Hepatic TAG declined from 1 to 8 wks on the WD and increased after 20 and 40 wks. After 40 wks on the WD hepatic TAG does not reach levels of TAG seen of LFD-fed female mice. Overall, this pattern of change in hepatic TAG and CHOL paralleled changes in MIS and MAS (**Fig 6A**).

Male mice show little change in the relative abundance of TAG, but a ~2-fold increase in CHOL over the first 8 wks of WD feeding (**Fig 6D).** After 20 and 40 wks on the WD, TAG increased ~1.7-fold and CHOL increased ~1.5-fold when compared to mice fed the LFD for 40 wks. Overall, this analysis revealed major differences in the type of steatosis (MIS versus MAS) and major neutral lipid classes (TAG and CHOL) during the 40-wk WD feeding period in female and male mice.

Admittedly, the decline in hepatic TAG and CHOL in female and male mice after 4 wks on the WD was unexpected. We are unaware of other investigators reporting this phenomenon. This is not likely an artifact since the results were seen in both female and male mice using two different methods, i.e., histological scoring (**Fig 6A and 6B**) and chemical analysis of neutral lipids (**Fig 6C & 6D**). We speculate these changes in hepatic neutral lipid storage may reflect changes in lipid partitioning between liver and adipose tissue over the course of WD feeding. Clearly, mice become insulin resistant by 4 wks on the WD which likely impacts lipid storage and mobilization in liver versus adipose tissue.

### Diet effects on liver injury

**Fig 7:**
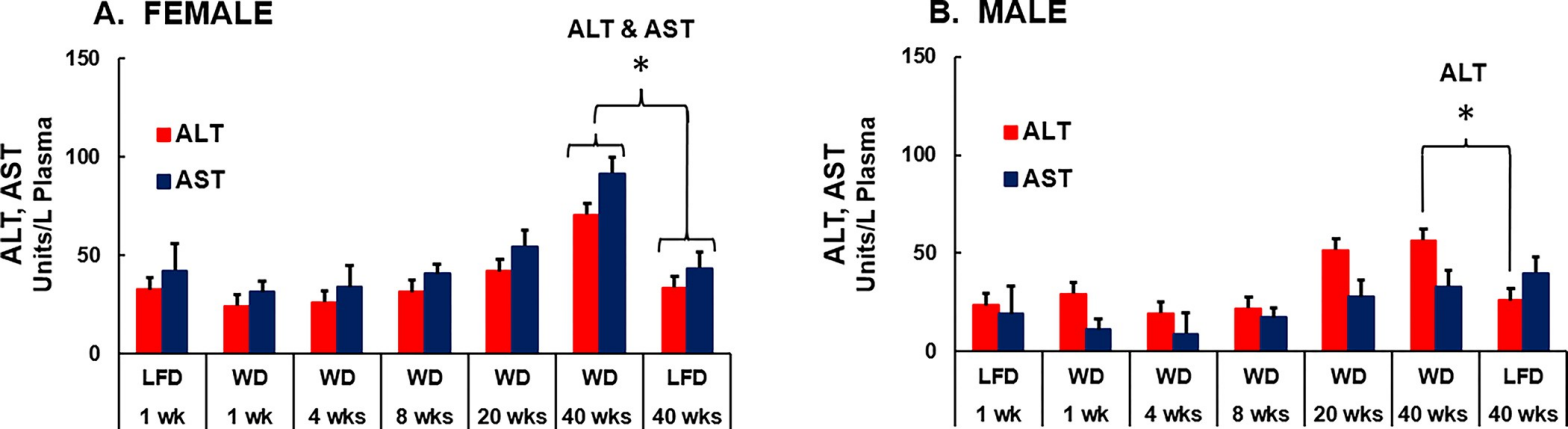
Time-course of WD effects on plasma markers of liver injury. Plasma levels of alanine aminotransferase (ALT) and aspartate aminotransferase (AST) in female [A] and male [B] *Ldlr*^*-/-*^ mice. Plasma levels of ALT and AST were measured as described in Methods. Statistical differences (p<0.05) between LFD and WD fed mice after 40 wks on the WD are designated by an asterisk (ӿ).

Evidence of WD-induced liver injury is illustrated in **Fig 7A & 7B.** Plasma levels of alanine amino transferase (ALT) and aspartate aminotransferase (AST) increased progressively from 8 to 40 wks on the WD in female mice. Plasma levels of both enzymes were significantly increased after 40 wks on the WD. In male mice, ALT, but not AST was significantly increased by the WD after 20 and 40 wks on the WD. These findings indicate that plasma markers of liver injury change well after WD-induced increases in insulin resistance (HOMA-IR, (**Fig 3**) and systemic inflammation (Tnfα, **Fig 4**), but parallel the increase hepatic MAS and neutral lipid storage after 20 wks on the WD (**Fig 6**).

### The western diet induces hepatic fibrosis

**Fig 8:**
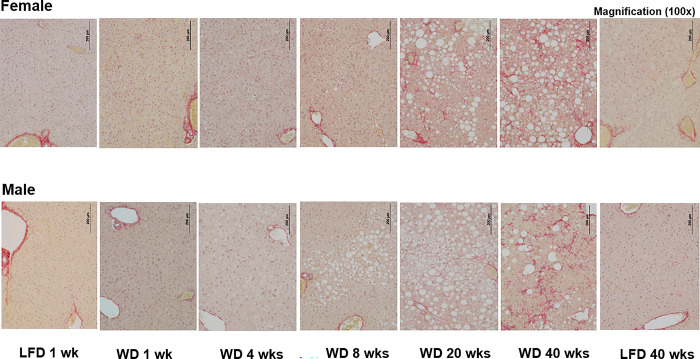
Time course of WD induced hepatic fibrosis. Representative histologic images of Picro Sirius Red (PSR) -stained liver from LFD and WD fed female and male *Ldlr*^*-/-*^ mice. Magnification: 100X.

**Fig 9:**
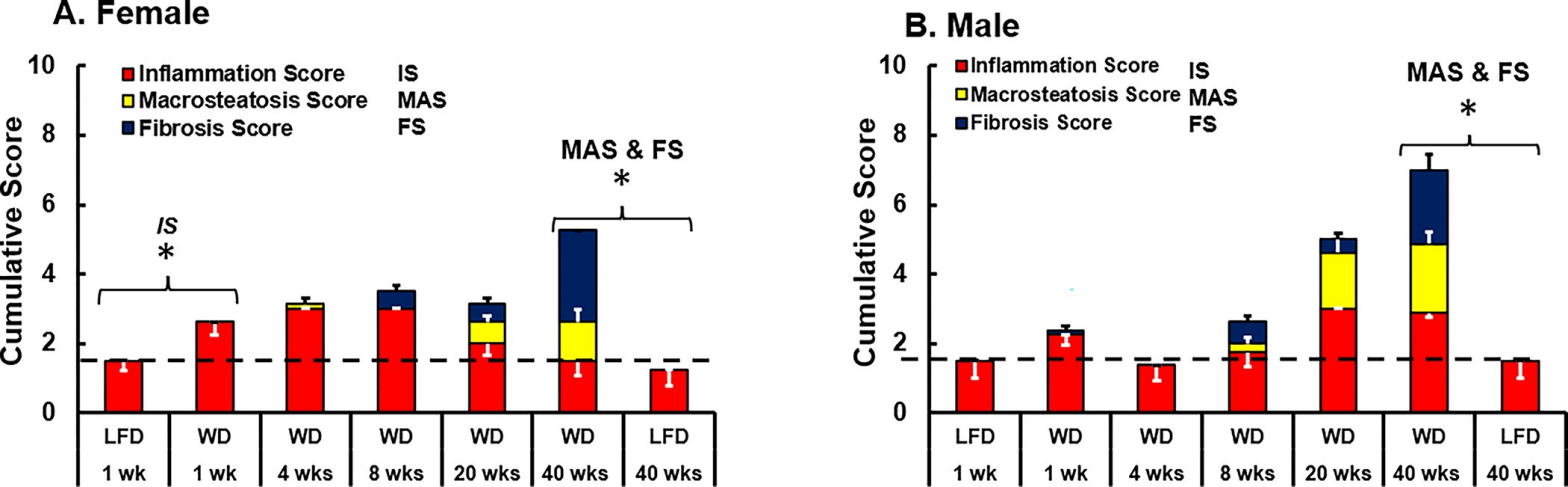
Time course of WD effects on hepatic inflammation, macrosteatosis and fibrosis. Histology scores for hepatic inflammation, macrosteatosis and fibrosis were used to prepare the stack bar graph. Results for female and male mice are shown in **Panels A** and **B**, respectively. Hepatic inflammation score (**IS**), macrosteatosis score (**MAS**) and fibrosis score (**FS**) were each scored on a 0 to 3-point scale. Results are presented as mean ± SEM. The statistical difference (p-value, < 0.05) for IS, MAS and FS between WD versus LFD groups at 1 and 40 wks is designated by an asterisk, (ӿ).

Hepatic fibrosis develops as a result of liver injury and liver injury increases after 8 wks on the WD (**Fig 7**). Picro Sirius Red (PSR) staining of liver slices was used to visualize collagen fibers (**Fig 8**). PSR staining is apparent in areas surrounding major hepatic vessels, including the portal triad composed of the hepatic artery, portal vein, bile duct, and central vein. Our studies show that WD-induced hepatic fibrosis develops in the centri-lobular/sinusoidal regions (**Figs 8 & S1D**) by 20 wks in mice fed the WD and worsens by 40 wks. Fibrosis scoring was carried out by veterinary pathologists as described in the Methods section and presented in **Fig 9**. Based on scoring, hepatic fibrosis increased in female and male mice after 8 wks on the WD (**Figs 8 & 9).**

In addition to steatosis and fibrosis, we also quantified hepatic inflammatory infiltrate to obtain the hepatic inflammation score (**Fig 9**). **S1B Fig** provides representative images of hepatic inflammation as observed in the H & E stained liver samples. We present the three histology scores (inflammation, steatosis and fibrosis) as stacked column plots to illustrate how these three parameters change over time in response to the WD. The dash line in **Fig 9** represents the basal level of inflammatory infiltrate in female and male mice. The inflammation score increased in both female and male mice 1.8- and 1.5-fold, respectively, after 1 wk on the WD. In female mice the inflammation score increased progressively to 8 wks. Afterward the inflammation score decreased to levels seen in mice fed the LFD for 40 wks. In male mice, the elevated inflammation score after 1 wk on the WD was not sustained at 4 wks, but was increased at 8, 20 and 40 wks on the WD. After 40 wks of WD feeding, the inflammation score was increased nearly 2-fold in male mice. Both steatosis and fibrosis are prominent in male and female mice after 20 wks on the WD.

In female mice, the increased hepatic inflammation score after 1 wk on the WD precedes the onset of MAS and fibrosis scores. This early inflammatory response of female mice to the WD coincides with the ~2-fold increase in plasma TNFα (**Fig 4A**). In male mice the hepatic inflammation score increased in parallel with the scores for MAS and fibrosis (**Fig 9A and 9B)**.

### Volcano plots reveal differential effects of the WD on female and male mice

**Fig 10:**
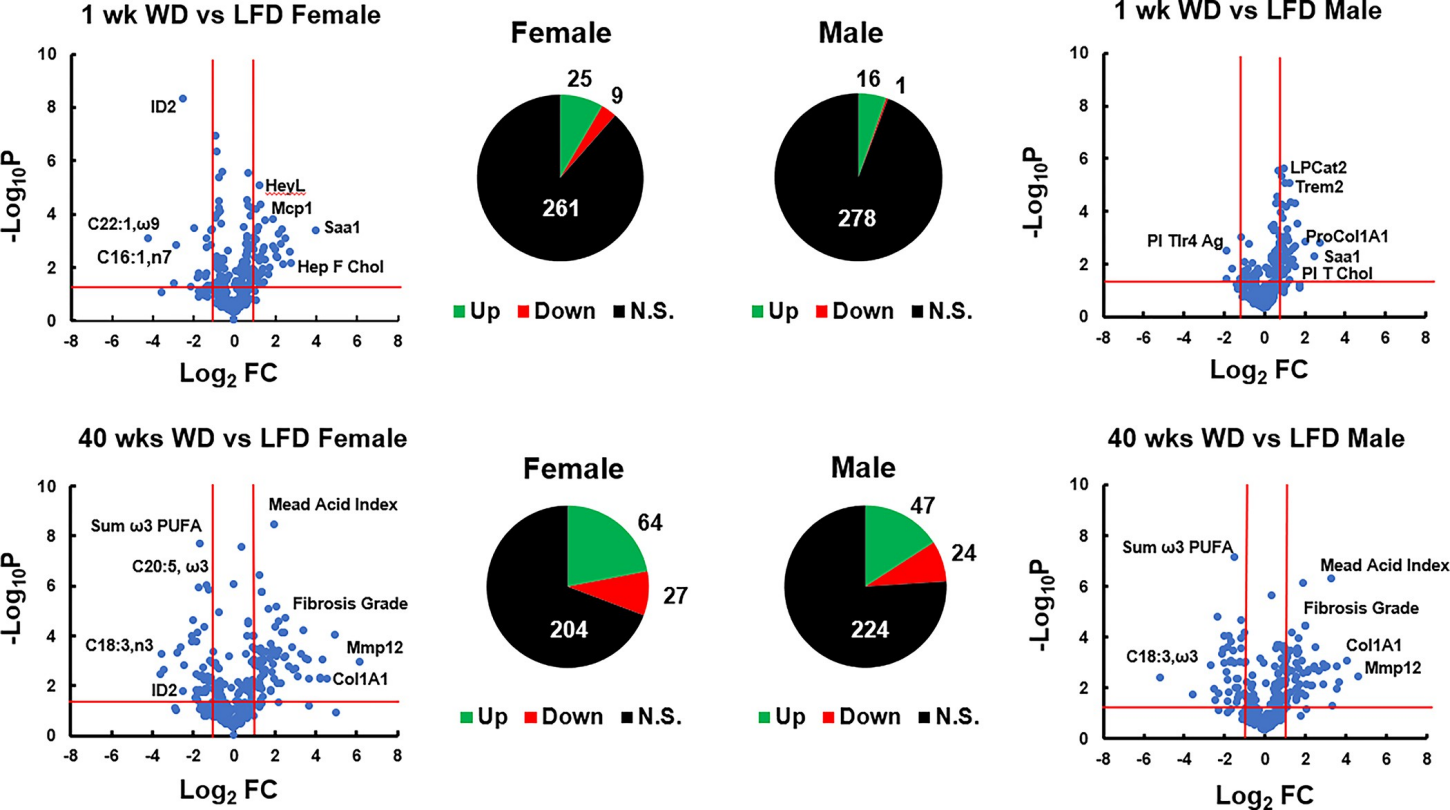
Volcano Plots of WD effects on anthropometric, plasma and hepatic markers. The comparisons were between LFD and versus WD fed mice at 1 wk and 40 wks. The graphs represent the -Log_10_ P-value (P) versus Log_2_ fold change (FC). The pie plots are a numerical summary of the markers that were increased (**UP**) or decreased (**Down**), or not significantly (**N.S**.) affected by the WD. All data used to prepare the volcano plots is in Supplementary Data: **S2 and S3 Tables**. A summary of the volcano plot data is presented in **S4 Table**.

A major goal of this study was to identify early markers that changed significantly in response to the WD that might play a role in NASH onset and progression. The preceding data analysis (**Figs 3–9**) has revealed a time course for onset and progression of major NASH markers, including the onset of insulin resistance, systemic inflammation, hepatic steatosis, inflammation and fibrosis in response to the WD. One outcome from this analysis is that female and male mice appear to have a different time-line for the response to the WD, particularly with respect to WD induced systemic and hepatic inflammation. To gain additional insight into the disease process, we used a statistical approach, i.e., volcano plots, to identify markers that responded rapidly to the WD in female and male mice. Overall, we assessed 295 markers **(S2 and S3 Tables),** including anthropometric, plasma and hepatic markers already discussed, as well as lipidomic (fatty acids, bile acid and oxylipins) and metabolomic (glutathione and NAD) and transcriptomic markers. The volcano and pie plots (**Fig 10)** represent a numerical assessment of markers responsive to feeding mice the WD for 1 wk and 40 wks.

After 1 wk on the LFD and WD, 25 and 16 markers were significantly increased, while 9 and 1 markers were significantly decreased in the WD fed group when compared to the LFD fed group in female and male mice, respectively. After 40 wks on the LFD and WD, 64 and 47 markers were significantly increased in female and male mice, while 27 and 24 markers were significantly decreased in WD versus LFD fed, respectively. These results add further support to the notion that female mice are more responsive to the WD than male mice.

Markers significantly induced in female mice after 1 wk on the WD included TLR agonists, hepatic fatty acids, bile acids, hepatic cholesterol, a microbial product (muramic acid, a gram-positive cell wall component) and multiple transcripts (**Table 1**). Male mice, in contrast, had fewer markers significantly induced after 1 wk on the WD. These markers included increases in several hepatic fatty acids, a plasma lipid marker and multiple transcripts.

**Table 1:** Markers induced or repressed by the western diet, [WD versus LFD] determined by the volcano plot analysis.

MARKERS INDUCED BY WD	Number of	Female	Female	Male	Male
**Marker Class Legend/ Method**	**Markers per Class**	**1 wk**	**40 wks**	**1 wk**	**40 wks**
Anthropometric (BW, LW, LW%BW)	6		1		1
Plasma Lipids, Assay	5		2	1	2
Plasma proteins, Assays	5		3		
Plasma, Glucose, Insulin, Homa-IR	3		2		2
Plasma TLR agonists, Assays	2	1	1		
Histology Scoring	5		3		2
Hepatic fatty acids/Gas Chromatography	34	5	5	3	6
Hepatic Bile Acids LC/MS	23	4	2		3
Hepatic lipids (Chol, TAG, etc.)	3	1	2		
Hepatic oxylipins, LC/MS	65				1
Oxidative stress, LC/MS	3		1		
Hepatic Microbial product, LC/MS	1	1	1		1
mRNA, qRTPCR	116	13	41	12	29
Hepatic non-esterified fatty acids, LC/MS	21				
Other metabolites (NAD, NADH), LC/MS	3				
**Total**	295	25	64	16	47
**MARKERS REPRESED BY WD**	**Number of**	**Female**	**Female**	**Male**	**Male**
**Marker Class Legend/Method**	**Markers per Class**	**1 wk**	**40 wks**	**1 wk**	**40 wks**
Anthropometric (BW, LW, LW%BW, % change in BW)	6				
Plasma Lipids, Assays	5				
Plasma proteins, Assays	5				
Plasma Glucose, Insulin, Homa-IR	3				
Plasma TLR agonist, Assays	2			1	
Histology scoring	5				1
Hepatic fatty acids/GC Chromatography	34	3	12		7
Hepatic Bile Acids, LC/MS	23		4		1
Hepatic lipids (Chol, TAG, etc.), Assays	3				
Hepatic oxylipins, LC/MS	65				
Oxidative stress, LC/MS	3	1	3		5
Microbial product, LC/MS	1				
mRNA, qRTPCR	116	5	8		10
Hepatic non-esterified fatty acids, LC/MS	21				
Other metabolites (NAD, NADH), LC/MS	3				
**Total**	295	9	27	1	24

Fewer markers were suppressed by the WD after 1 wk on the WD. In female mice, these markers included some hepatic fatty acids, the hepatic glutathione status, i.e., (reduced / oxidized (GSH/GSSG) ratio and several mRNA transcripts. In male mice, only TLR4-Ag were suppressed by the WD within 1 wk. Taken together, these results indicate that female and male mice respond differently to the WD.

**Fig 11:**
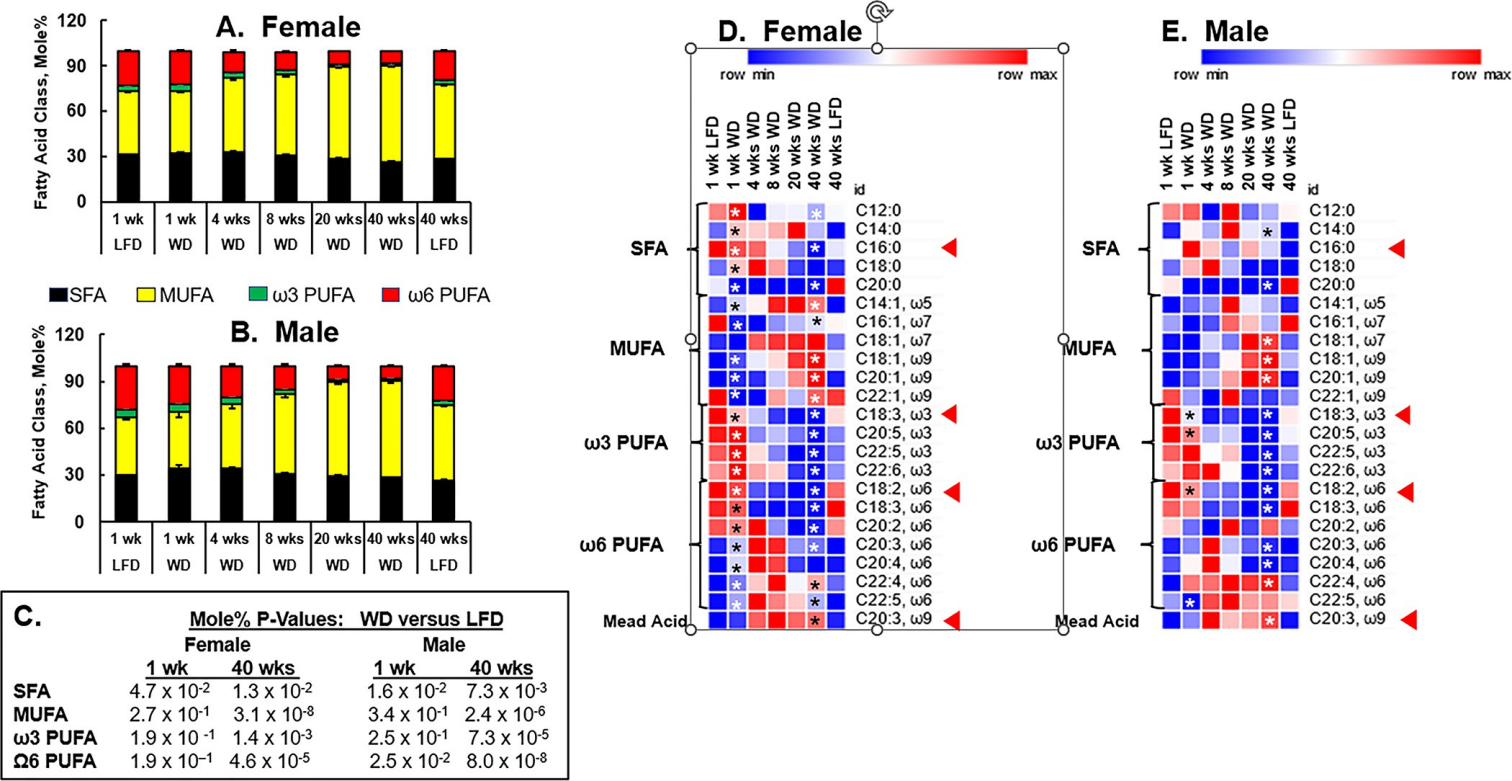
Time course for WD effects on hepatic fatty acid content in female and male *Ldlr*^*-/-*^ mice. Total hepatic lipids were extracted, saponified, methylated and fractionated by gas chromatography as described in Methods. **A & B:** The stacked bar graphs represent the mole% ± SEM of hepatic fatty acids in each of the four fatty acid classes: Saturated fatty acids (SFA), monounsaturated fatty acids (MUFA), ω3 polyunsaturated fatty acids (ω3 PUFA) and ω6 polyunsaturated fatty acids (ω6 PUFA). **C:** The p-values for the data presented in panels A and B. **D and E** Heat Maps: Results are presented as the mean of the mole% for each fatty acid, normalized to hepatic protein (μmoles/g protein), at each time point. Specific fatty acids (16:0, 18:2, ω6 & 18:3, ω3, 20:3, ω9)) are marked by a red arrow (◄). Data for these fatty acids are graphed in **Fig 12** to show precursor-product relationship. An asterisk (ӿ) in the heat maps designates statistical differences (p<0.05) between WD versus LFD after 1 and 40 wks on the diets.

**Fig 12:**
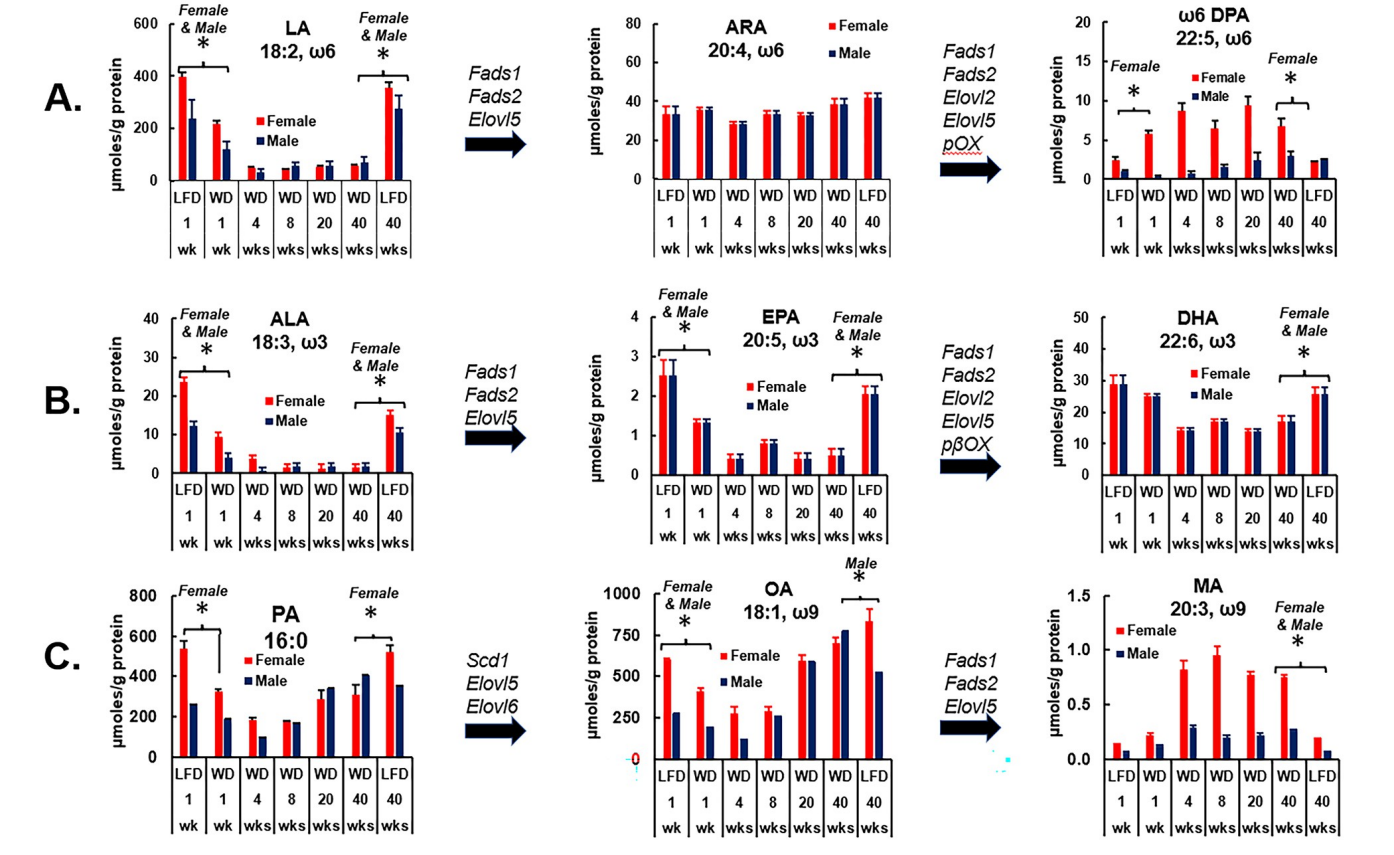
Time course for WD effects on hepatic abundance of LA (18:2, ω6), ALA (18:3, ω3) and PA (16:0) and the elongation and desaturation products derived from these fatty acids. Hepatic fatty acids were extracted and fractionation as described in Methods and **Fig 11**. Results are expressed as μmoles fatty acids/g hepatic protein (μmoles/g protein). **Panel A**: linoleic acid (LA), arachidonic acid (ARA) & ω6-docosapentaenoic acid (ω6 DPA, 22:5, ω6). **Panel B**: α-linolenic acid (ALA), eicosapentaenoic acid (EPA) and docosahexaenoic acid (DHA); **Panel C**: palmitic acid (PA), oleic acid (OA) and mead acid (MA). Fads1, fatty acid desaturase 1; Fads2, fatty acid desaturase 2; Elovl2, fatty acid elongase 2, Elovl5 fatty acid elongase 5, pβOx, peroxisomal fatty acid β-oxidation. Significant differences (p< 0.05) between WD versus LFD groups at 1 and 40 wks are designated by an asterisk (ӿ).

**Fig 13:**
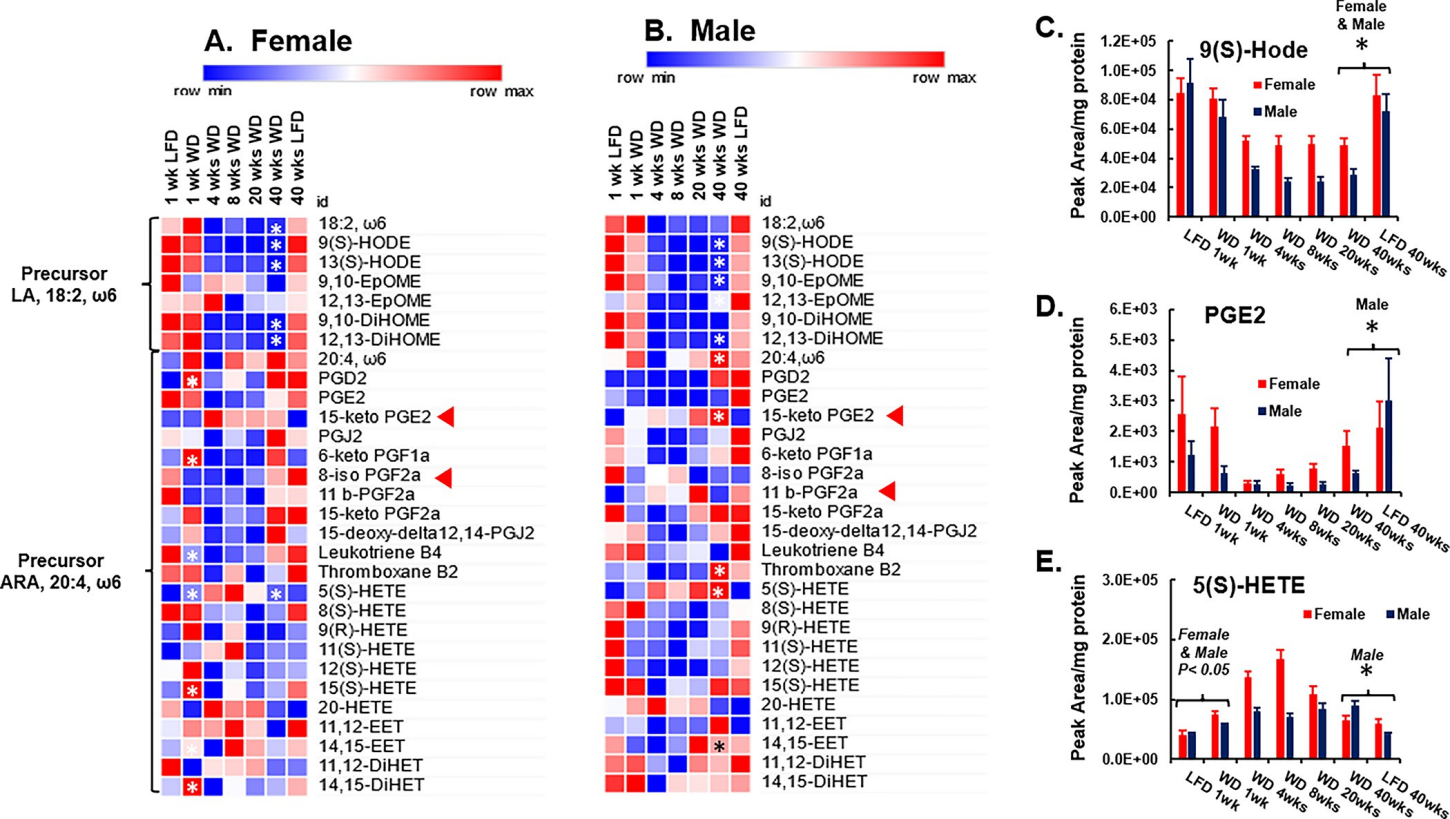
Time course of WD effects hepatic ω6 PUFA-derived oxylipins. **A and B:** The heat map represents the average abundance of non-esterified ω6-fatty acids (18:2, ω6 and 20:4, ω6) and oxylipins derived from these fatty acids normalized to hepatic protein, i.e., Peak area/mg hepatic protein. 18:2,ω6, LA, linoleic acid; 9(S)-HODE, 9S-hydroxy-10E,12Z-octadecadienoic acid; 13(S)-HODE, 13S-hydroxy-9Z,11E-octadecadienoic acid; 9,10-EpOME, (±)9,10-epoxy-12Z-octadecenoic acid; 12,13-EpOME, 12(13)epoxy-9Z-octadecenoic acid; 9,10-DiHOME, 9,10-dihydroxy-12Z-octadecenoic acid; 12,13-DiHOME, 12,13-dihydroxy-9Z,octadecenoic acid; 20:4,ω6, ARA, arachidonic acid; PGD2, prostaglandin D2; PGE2, prostaglandin E2; 15-Keto PGE2, 15 keto prostaglandin E2; PGJ2, prostaglandin J2; 6-keto PGF1α, 6-keto prostaglandin F1α-d4; 8-iso PGF2α, 8-iso prostaglandin F2α; 11 β-PGF2α: 11-Epi-prostaglandin F2α; 15-keto PGF2α, 15-keto-prostaglandin F2α; 15-deoxy-Δ12,14-PGJ2, 15-Deoxy-Δ12,14-prostaglandin J2; Leukotriene B4; Thromboxane B2; 5(S)-HETE: 5-Hydroxyeicosatetraenoic acid; 8(S)-HETE: 8-hydroxyeicosa-tetraenoic acid; 9(R)-HETE: 9R-hydroxy-5Z,7E,11Z,14Z-eicosatetraenoic acid; 11(S)-HETE, 11S-hydroxy-5Z,8Z,12E,14Z-eicosatetraenoic acid; 12(S)-HETE: 12S-hydroxy-5Z,8Z,10E,14Z-eicosatetraenoic acid; 15(S)-HETE: 15-Hydroxyeicosatetraenoic acid; 20-HETE: 20-Hydroxyeicosatetraenoic acid; 11,12-EET: 11,12-Epoxyeicosatrienoic acid; 14,15-EET: 14,15-Epoxy-5Z,8Z,11Z-eicosatrienoic acid; 11,12-DiHET: 11,12-Dihydroxy-5Z,8Z,14Z-eicosatrienoic acid; 14,15-DiHET: 14,15-Dihydroxy-5Z,8Z,11Z-eicosatrienoic acid. Statistical differences (p < 0.05) between LFD and WD fed mice at 1 and 40 wks are represented by an asterisk ӿ). The red arrow (◄) in the heat maps point to an inactive metabolite of Pge2, 15-keto Pge2 and a marker of lipid peroxidation, 8-iso PGF2α. Statistical significance (p < 0.05) at 1 and 40 wks for WD versus LFD-fed mice is designated by (ӿ). **C–D:** Graphs for LA- and ARA-derived oxylipins: **C.** 9(S)-HODE, **D.** PGE2 and **E.** 5(SHETE) are presented as peak area/mg hepatic protein. Statistical significance (p < 0.05) at 1 and 40 wks for WD versus LFD is designated by an asterisk (ӿ).

**Fig 14:**
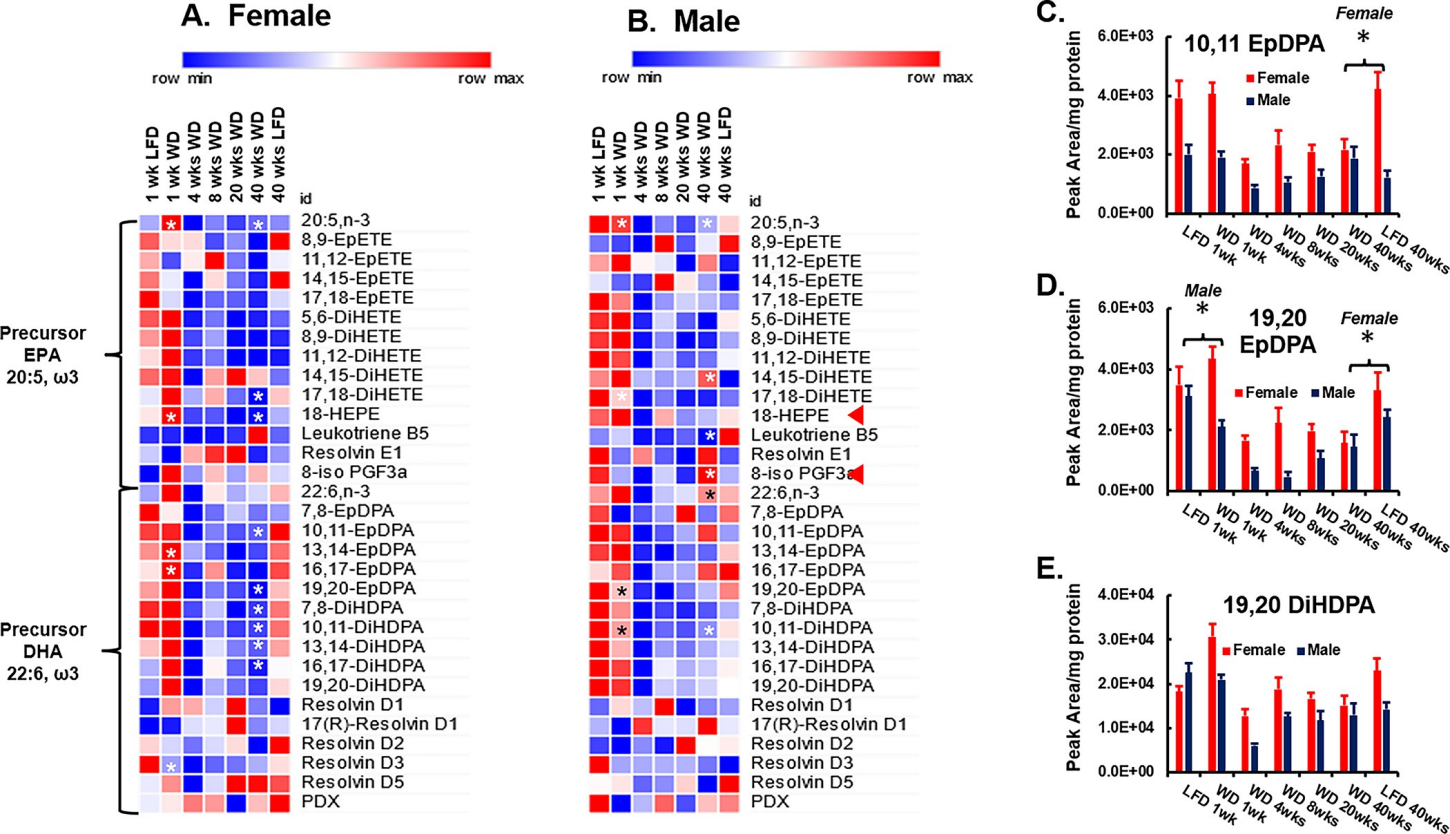
Time course of WD effects on hepatic ω3 PUFA-derived oxylipins. **A & B:** The heat map represents the average abundance of non-esterified ω3-fatty acids (20:5, ω3 and 22:6, ω3) and oxylipins derived from these fatty acids normalized to hepatic protein, i.e., Peak area/mg hepatic protein. The methods used to extract and analyze hepatic oxylipins is described in the Materials and Methods. 20:5, ω3: Eicosapentaenoic acid; 8,9-EpETE: (5Z,11Z,14Z,17Z)-8,9-epoxyicosatetraenoic acid; 11,12-EpETE: (5Z,8Z,14Z,17Z)-11,12-epoxyicosatetraenoic acid; 14,15-EpETE: (±)14,15-epoxy-5Z,8Z,11Z,17Z-eicosatetraenoic acid; 17,18-EpETE: (±)17,18-epoxy Eicosatetraenoic Acid; 5,6-DiHETE: (±)5,6-dihydroxy-8Z,11Z,14Z,17Z-eicosatetraenoic acid; 8,9-DiHETE: 8,9-dihydroxy-5Z,11Z,14Z,17Z-eicosatetraenoic acid; 11,12-DiHETE: 11,12-dihydroxy-5Z,8Z,14Z,17Z-eicosatetraenoic acid; 14,15-DiHETE: (±)14,15-dihydroxy-5Z,8Z,11Z,17Z-eicosatetraenoic acid; 17,18-DiHETE: (±)17,18-dihydroxy-5Z,8Z,11Z,14Z-eicosatetraenoic acid; 18-HEPE: (±)-18-hydroxy-5Z,8Z,11Z,14Z,16E-eicosapentaenoic acid; Leukotriene B5: 5S,12R-dihydroxy-6Z,8E,10E,14Z,17Z-eicosapentaenoic acid; 8-iso PGF3α, 8-iso prostaglandin F3α; DHA: Docosahexaenoic acid; 7,8-EpDPA: (±)7,8-epoxy Docosapentaenoic Acid; 10,11-EpDPA: (±)10,11-epoxy docosapentaenoic acid; 13,14-EpDPA: (±)13,14-epoxy Docosapentaenoic Acid; 16,17-EpDPA: (±)16,17-epoxy Docosapentaenoic Acid; 19,20-EpDPA: (±)19,20-epoxy Docosapentaenoic Acid; 7,8-DiHDPA: (±)7,8-dihydroxydocosa-4Z,10Z,13Z,16Z,19Z-pentaenoic acid; 10,11-DiHDPA: (±)10,11-dihydroxy-4Z,7Z,13Z,16Z,19Z-docosapentaenoic acid; 13,14-DiHDPA: 13,14-dihydroxy-4Z,7Z,10Z,16Z,19Z-docosapentaenoicacid; 16,17-DiHDPA: (±)16,17-dihydroxy-4Z,7Z,10Z,13Z,19Z-docosapentaenoic acid; 19,20-DiHDPA: (±)19,20-dihydroxy-4Z,7Z,10Z,13Z,16Z-docosapentaenoic acid; Resolvin: 17R resolvin D1; Resolvin D1; Resolvin D2; Resolvin D3; Resolvin D5; PDX, Protectin DX. Statistical differences between LFD and WD fed mice at 1 and 40 wks is represented by an asterisk (*) p < 0.05 1 wk WD versus 1 wk LFD and/or 40 wks WD versus 40 wks LFD. The red arrows (◄) in the heat maps point to EPA-derived non-enzymatically oxidized lipids, i.e., 18-HEPE and 8-isoPGFα3. **C–E:** DHA-derived oxylipin data for **C:** 10, 11 EpDPA, **D:** 19,20 DpDPA and **E:** 19,20 DiHDPA is presented in the figure graphically and reported as peak area/mg hepatic protein. Statistical significance (p < 0.05) at 1 and 40 wks for WD versus LFD is designated by an asterisk (ӿ).

**Fig 15:**
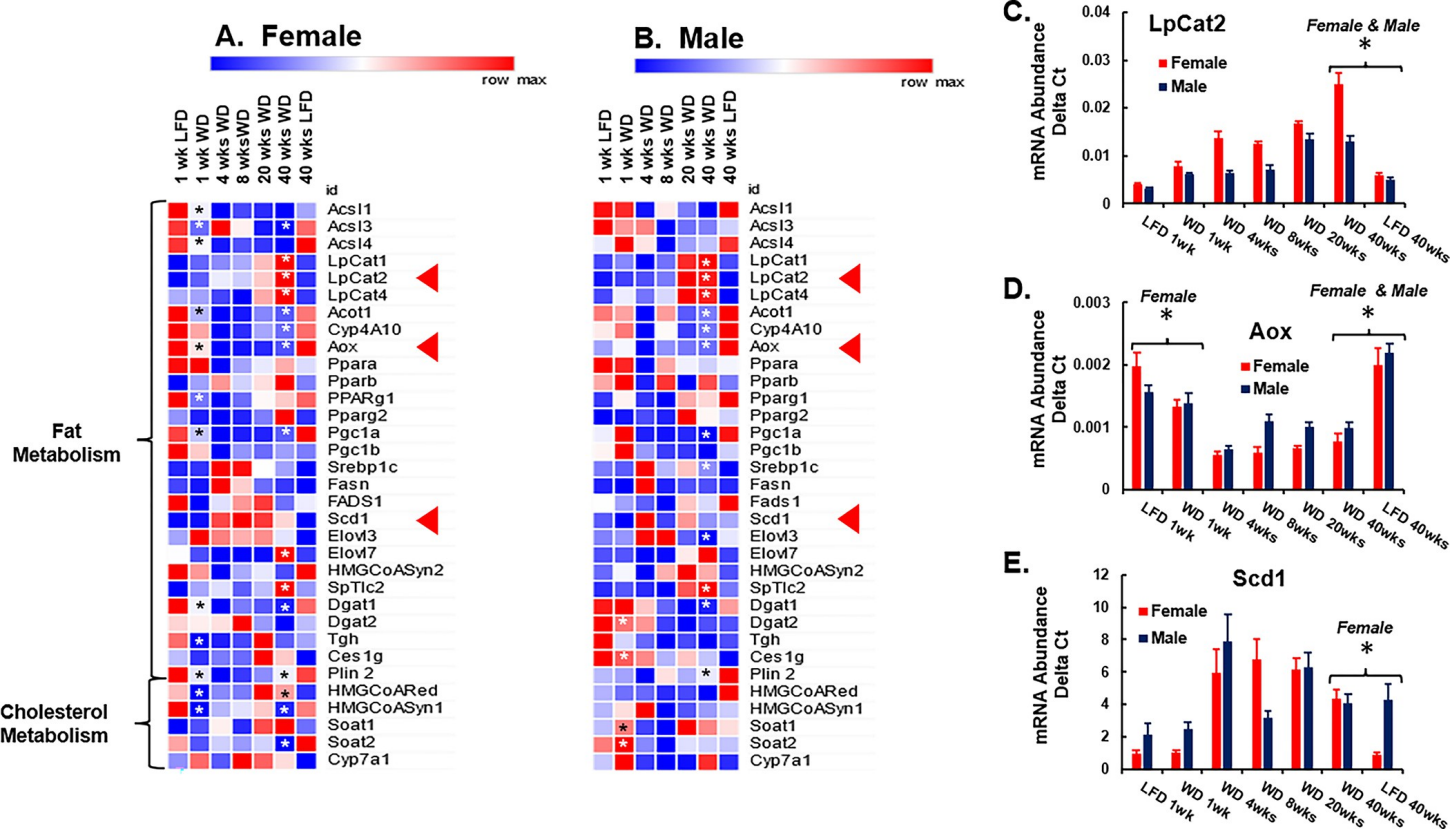
WD effects on hepatic gene expression: Lipid metabolism. Hepatic mRNA abundance was quantified by qRTPCR. **A. & B.** Results in the heat maps are presented as the mean value for mRNA abundance. **C -E.** Results in graphs are presented as the mean ± SEM of mRNA abundance-delta C_T_. The following transcripts were quantified: Acsl, Acyl CoA synthetase long chain; LpCat, Lysophosphatidylcholine acyl transferase; Acot1, Acyl CoA thioesterase-1; Cyp4A10, Cytochrome P450 4A10; Aox, Acyl CoA oxidase; PPAR (α, β, γ1, γ2], peroxisome proliferator activated receptor; PGC (1α, 1β) PPARγ co-activator; Srebp1c, Sterol regulatory element binding protein 1c; Fasn, Fatty acid synthase, Fads1, Fatty acid desaturase 1; Scd1, Stearoyl CoA desaturase 1; Elovl3, Fatty acid elongase 3, HmgCoASyn2, Hydroxymethylglutaryl-CoA synthase 2; SpTlc2, Serine palmitoyltransferase, long chain base subunit 1; Dgat (1, 2), Diacylglycerol acyl transferase; Tgh, Triglyceride hydrolase; Ces1g, Carboxyesterase 1g; Plin2, Perilipin 2; HMGCoARed, Hydroxymethylglutaryl-CoA reductase; HmgCoAsyn1, Hydroxymethylglutaryl-CoA synthase 1; Soat, Sterol-O-acyl transferase; Cyp7a1, Cytochrome P450 7A1. Significant differences (p-value (p < 0.05) between WD versus LFD groups after 1 and 40 wks in the heat maps is designated by an asterisk (ӿ). The arrows (◄) in the heat maps point to transcripts that were graphed, and include C: LpCat2, D: Aox and E: Scd1. Results in the graphs are reported as mRNA Abundance Delta C_T_. Statistical differences (p<0.05) between WD versus LFD at 1 and 40 wks is designated by an asterisk (ӿ).

**Fig 16:**
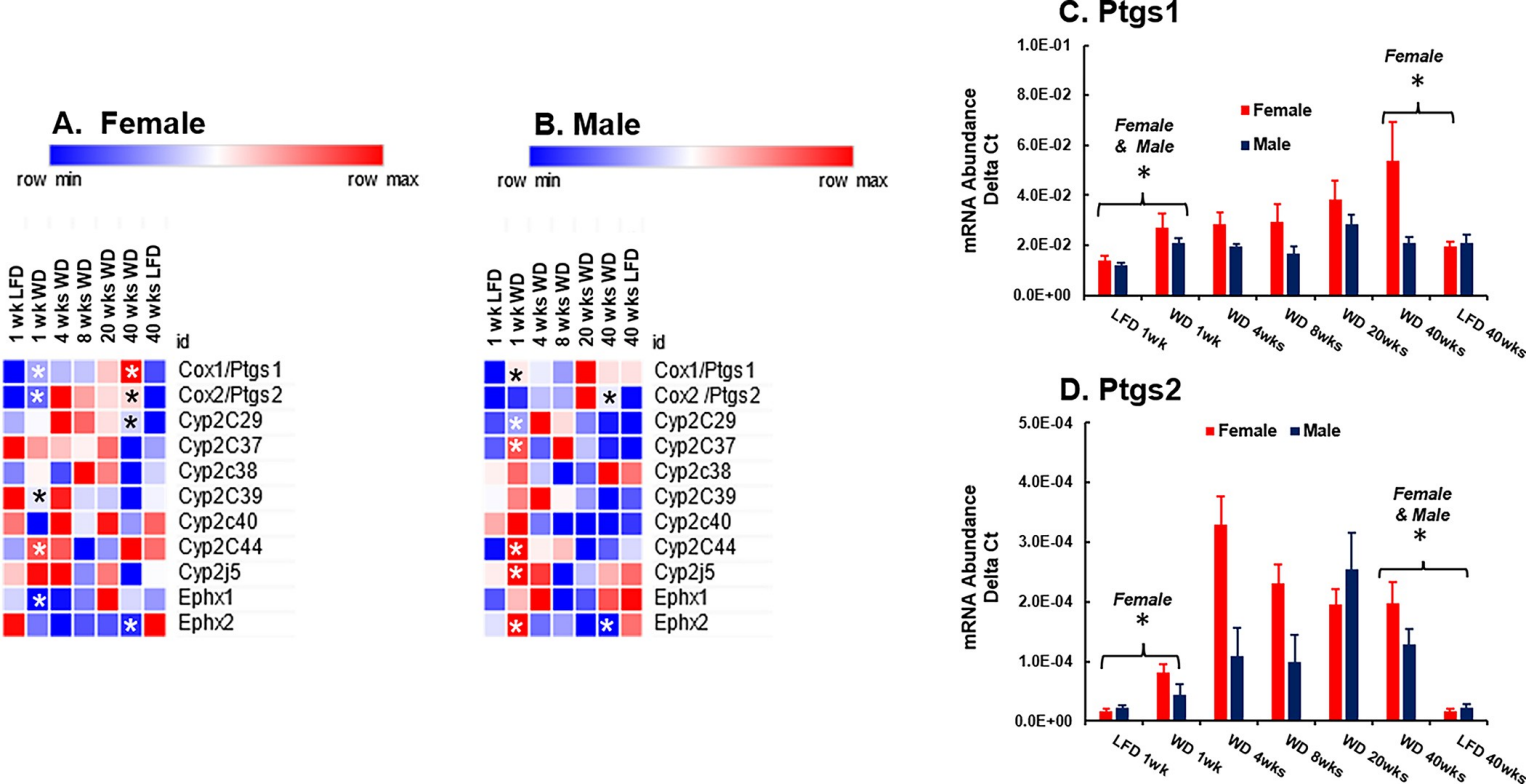
WD effects on hepatic gene expression: Oxylipin metabolism. Hepatic mRNA abundance was quantified by qRTPCR. **A & B:** Results in the heat maps are presented as the mean value for mRNA abundance. **C & D:** Results are presented as the mean ± SEM of mRNA abundance-delta C_T_. The transcripts examined include prostaglandin synthase (Ptgs 1 & Ptgs 2), the epoxygenases: Cytochrome P450 CYP2C subtypes 29, 37, 38, 39, 40, 44, Cyp2J5 and the epoxide hydrolases, Ephx1 and Ephx2. Heat maps: Significant differences (p-value; p < 0.05) between WD versus LFD groups after 1 and 40 wks is designated by an asterisk (ӿ), Graphs Ptgs1 and Ptgs2 statistical differences (p<0.05) between WD versus LFD groups at 1 and 40 wks is designated by an asterisk (ӿ).

**Fig 17:**
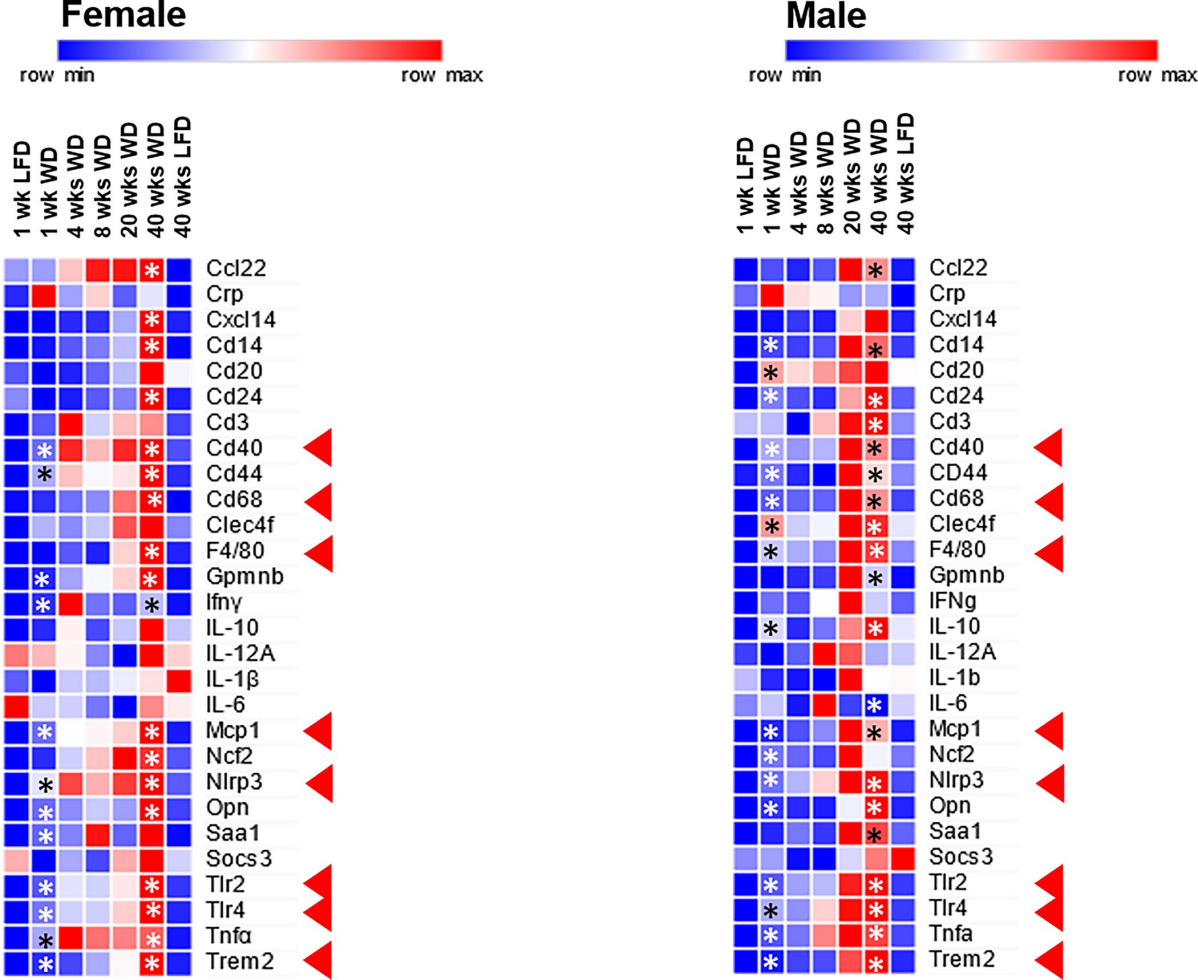
WD effects on hepatic gene expression: Inflammation-1. Hepatic mRNA abundance was quantified by qRTPCR. Results are presented as the mean value for mRNA abundance. The transcripts examined include: **Cd22**, cluster of differentiation 22; **Crp**, C-reactive peptide; **Cxcl14**, C-X-C motif chemokine ligand 14; **Cd14**, cluster of differentiation 14; **Cd20**, cluster of differentiation 20; **Cd24**, cluster of differentiation 24; **Cd3**, cluster of differentiation 3; **Cd40**, cluster of differentiation 40; **Cd44**, cluster of differentiation 44; **CD68**, cluster of differentiation 68; **Clec4f**, C-type lectin domain family 4 member F; **F4/80 (Adgref4)**, adhesion G-protein coupled receptor F4; **Gpnmb**, glycoprotein nmb; **IFNγ**, interferon γ; **IL10**, interleukin 10; **IL12A**, interleukin 12A; **IL1β**, interleukin 1β; **IL6**, Interleukin 6; **Mcp1**, monocyte chemoattractant protein 1; **Ncf2**, neutrophil cytosolic factor 2; **Nlrp3**, NLR family pyrin domain containing 3 (inflammasome); **Opn**, osteopontin; **Saa1**, serum amyloid A1; **Socs3**, suppressor of cytokine signaling 3; **TLR2**, toll-like receptor 2; **TLR4**, Toll-like receptor 4; **TNFα**, tumor necrosis factor α; **Trem,2,** triggering receptor expressed on myeloid cells. The red arrows (◄) in the heat maps point to transcripts that are graphed in Fig 18. Results are presented as the mean of mRNA abundance. Significant differences (p-value, p < 0.05) between the comparison of WD versus LFD groups after 1 and 40 wks is designated by an asterisk (ӿ).

**Fig 18:**
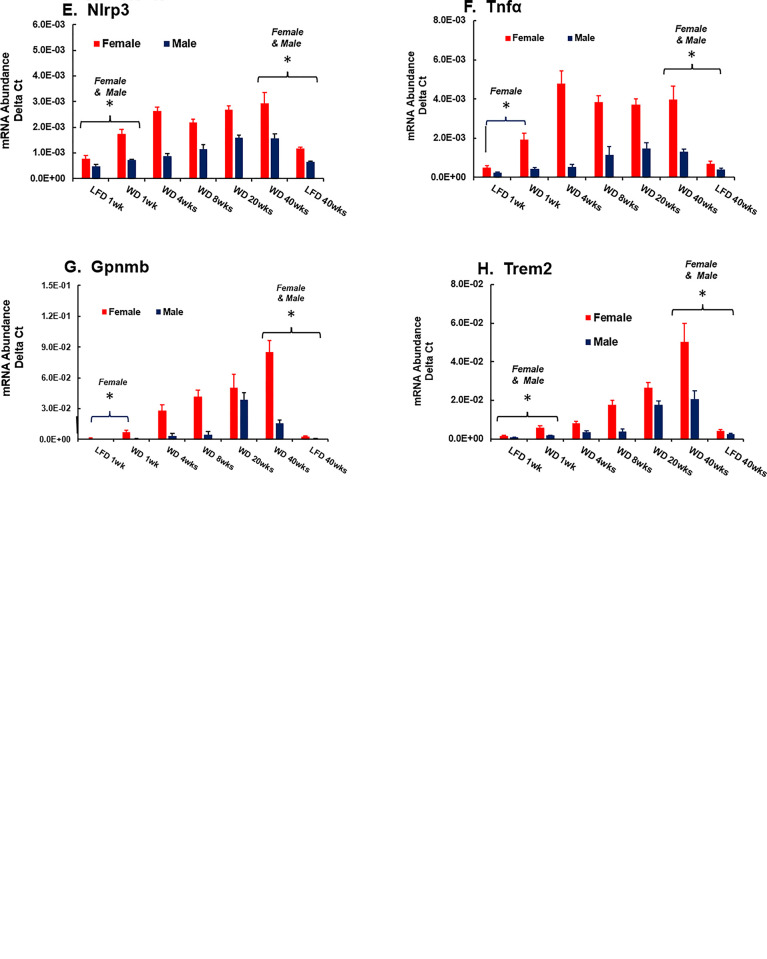
WD effects on hepatic gene expression: Inflammation-2. Hepatic mRNA abundance was quantified by qRTPCR. The results are presented as the mean ± SEM of mRNA abundance-delta C_T_. The transcripts examined include: **A**. Mcp1, monocyte chemoattractant protein; **B.** CD40, cluster of differentiation 40; **C.** CD68, cluster of differentiation 68**; D.** F4/80, adhesion G-protein coupled receptor F4; **E.** Nlrp3, NLR family pyrin domain containing 3 (inflammasome); **F.** Tnfα, tumor necrosis factor α, **G**, Gpnmb, glycoprotein nmb; **H.** Trem,2, triggering receptor expressed on myeloid cells. Statistical differences (p<0.05) between WD versus LFD groups at 1 and 40 wks are designated by an asterisk (ӿ).

**Fig 19:**
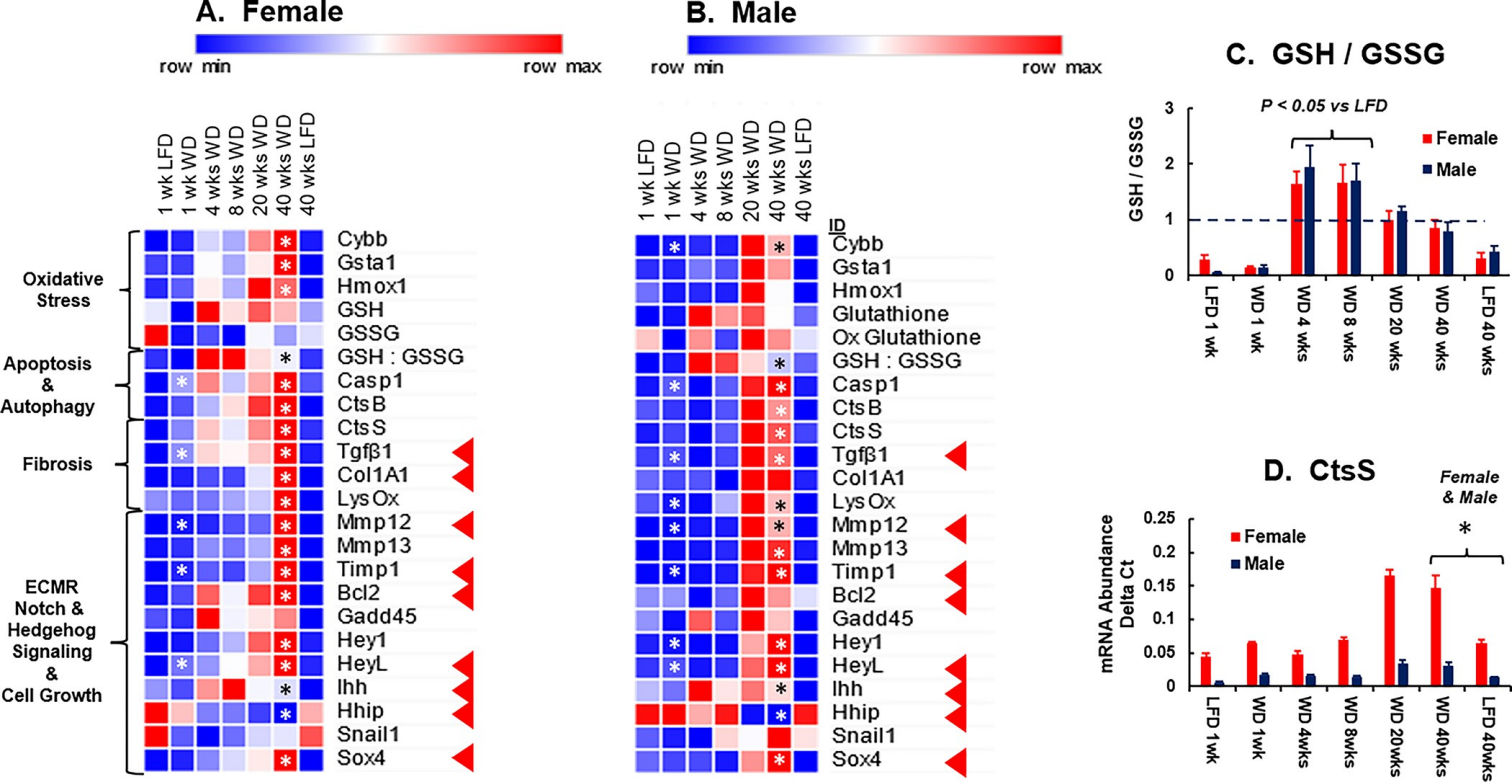
WD effects on hepatic gene expression: Oxidative stress, fibrosis, extracellular matrix remodeling and cell growth. Hepatic mRNA abundance was quantified by qRTPCR. **A and B:** Results in the heat maps are presented as the mean value for mRNA abundance of each transcript. The transcripts presented in the heat maps include Cybβ, cytochrome B245 β-chain; Gstα1, glutathione S transferase α1; Hmox1, heme oxygenase 1; Casp1, caspase 1; CtsB, cathepsin B; CtsS, cathepsin S; Col1A1, collagen 1A1; LysOx, lysyl oxidase; Tgfβ1, transforming growth factor β1; Mmp12, matrix metalloprotein 12; Mmp13, matrix metalloprotein 13, Timp1, tissue inhibitor of metalloproteases 1; Bcl2, B-cell lymphoma 2; Gadd45, growth arrest and DNA damage inducible; Gpc3, glypican 3; Hey1, Hes family bHLH transcription factor with YRPW motif1; HeyL, Hes family bHLH transcription factor with YRPW motif 1-like; Ihh, Indian hedgehog; Hhip, hedgehog interacting protein; Snail1, snail family transcriptional repressor; Sox4, SRY-box transcription factor 4. In the heat maps, statistical significance (p-value, p<0.05) between WD versus LFD groups at 40 wks is designated by asterisk (ӿ). The red arrows (◄) in the heat map point to transcripts that will be graphed in Fig 20. **C:** Ratio of hepatic reduced glutathione (GSH) and oxidized glutathione (GSSG): GSH/GSSG. The results are reported as the mean ± SEM. The “dash” line in the graph indicates that hepatic levels of GSH and GSSG are equal. In the GSH/GSSG graph, statistical significance between values at 4 and 8 wks on the WD versus 1 and 40 wks on the LFD is designated by an asterisk (ӿ). **D**: the mRNA abundance for cathepsin S (CtsS) is presented as mRNA Abundance Delta Ct, mean ± SEM. Statistical differences in CtsS mRNA abundance after 40 wks on the WD versus LFD is designated by an asterisk (ӿ).

**Fig 20:**
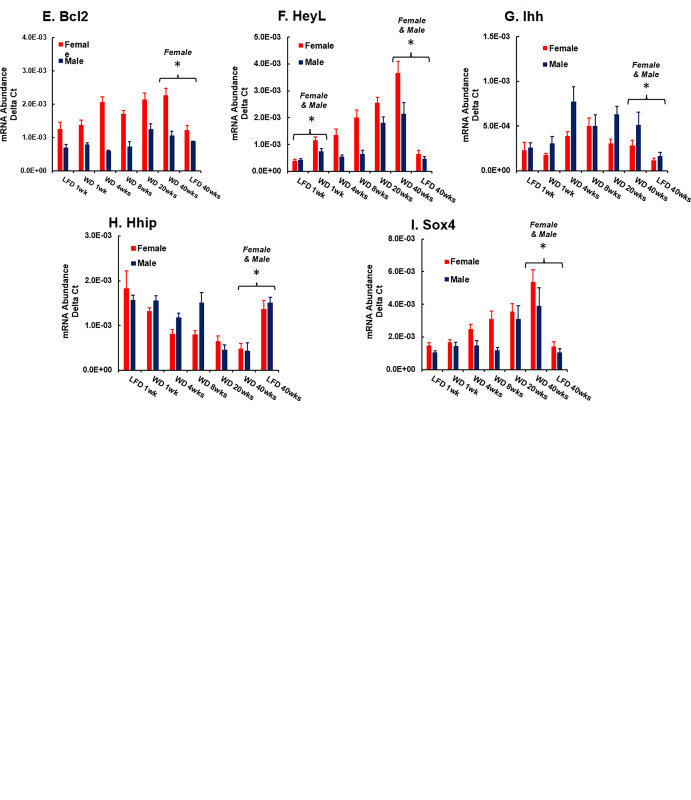
WD effects on the expression of hepatic transcripts involved in fibrosis, extracellular matrix remodeling and cell growth. Hepatic mRNA abundance results are presented as the mean ± SEM for mRNA abundance Delta C_T_. The transcripts examined include: A. Col1A1 (collagen 1A1), B. Tgfβ1 (transforming growth factor β1), C. Mmp12 (matrix metalloprotease 12), D. Timp1 (tissue inhibitor of metalloproteases 1), E. Bcl1 (B-cell lymphoma 2), F. HeyL (Hes-related family bHLH transcription factor with YRPW motif-like), G. Ihh (Indian hedgehog), H. Hhip (hedgehog interacting protein Hhip) and I. Sox4, RY-box transcription factor 4). Statistical differences (p < 0.05) between WD versus LFD fed mice after 1 and 40 wks are designated by an asterisk (ӿ).

**Fig 21:**
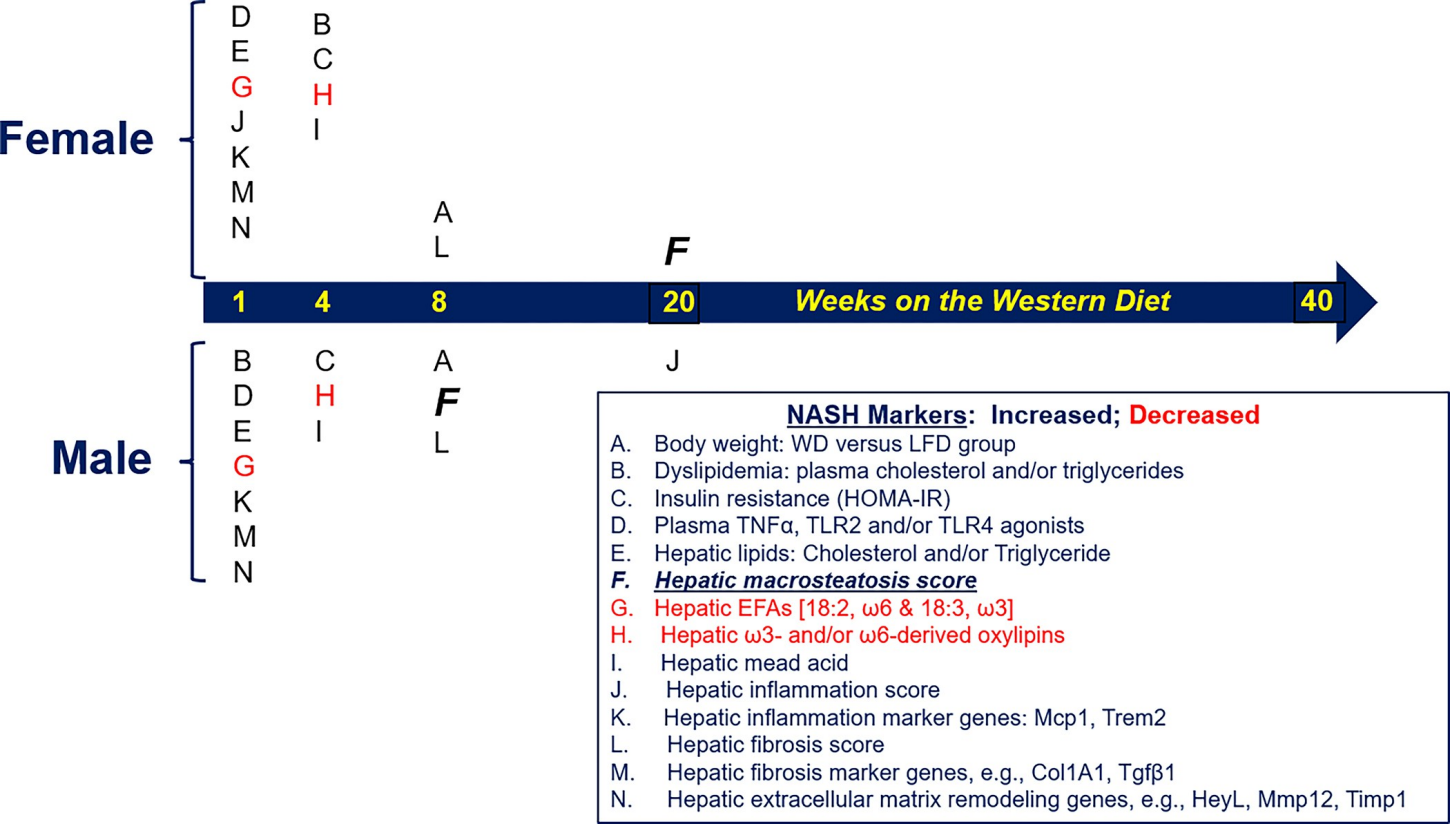
Summary of the earliest time point for significant WD-induced changes in NASH-associated markers. The table list 14 NASH markers that increased or decreased in response to feeding female and male mice the western diet (WD). The figure illustrates the earliest time point for a significant change in the marker abundance and whether the marker **Increased** or **Decreased** in response to the WD. This assessment is based on the data presented in **Figs 2–20**.

In **Figs 11–21** we focus our analysis on WD-induced changes in hepatic fatty acids, oxylipins and gene expression markers of NASH. We present the data in both graphs and heat maps. The graphs were constructed using the mean ± SEM, while the heat maps were constructed using the mean value of the marker assessed. Both data presentations used all data for diet (LFD and WD) and all time points (i.e., 1, 4,8, 20 and 40 weeks [wks]) in the analysis. Statistical differences (p-value, p < 0.05) between WD versus LFD at 1 and 40 wks are indicated in the graphs and heat maps by an asterisk (_**ӿ**_).

### The WD rapidly alters hepatic fatty acid composition

As previously reported, the hepatic fatty acid profile is significantly affected by feeding *Ldlr*^*-/-*^ mice the WD for several months [43,47]. In this report we determined how quickly the WD affected hepatic fatty acid content. Accordingly, hepatic lipid was extracted, saponified, methylated and fractionated by gas chromatography as described in Methods. This approach allows for the identification and quantitation of fatty acids in ester linkage in complex lipids, as well non-esterified fatty acids. We present the fatty acid data as stacked column plots to illustrate how the WD affected the mole% of the 4 major fatty acid classes, i.e., saturated fatty acids (SFA), monounsaturated fatty acids (MUFA), ω3 polyunsaturated fatty acids (PUFA) and ω6 PUFA **(Fig 11A & 11B)**. The p-values for the diet effects (WD versus LFD) for SFA, MUFA, ω3 PUFA and ω6 PUFA are presented in **Fig 11C**.

MUFA represent the predominant fatty acid class in liver; the rank order is MUFA > SFA > ω6 PUFA > ω3 PUFA. The WD had minor effects on the overall mole% of SFA, but increased the mole% of hepatic MUFA throughout the 40 wk WD feeding period. In contrast, the mole % of both ω3 PUFA and ω6 PUFA decreased in response to the WD over the same time frame. Thus, feeding mice the WD enriched liver fat with SFA and MUFA and lowered hepatic ω3 and ω6 PUFA. This pattern of change in hepatic fatty acid class in response to the WD was seen in both female and male mice and this outcome confirmed our previous findings on WD effects on liver lipids in mice [43,44].

The heat maps (**Fig 11D & 11E**) provide a detailed view of WD effects on the mole% of 23 hepatic fatty acids in the 4 fatty acid classes, SFA, MUFA, ω3 PUFA and ω6 PUFA. In addition, we also included mead acid (MA, 20:3, ω9) in the analysis. The WD significantly affected the hepatic content of nearly all fatty acids in livers of female and male mice over the 40 wk feeding period. Particularly relevant is the decline in the essential fatty acids (EFAs), linoleic acid (LA, 18:2, ω6) and α-linolenic acid, (ALA, 18:3, ω3) within 1 wk on the WD in both the female and male mice. The red arrows (◄) in **Fig 11D & 11E** point to LA and ALA. The significant decline in LA and ALA after 1 wk on the WD was associated with a decline in multiple, but not all, C_18-22_ ω6 and ω3 PUFA derived from LA and ALA, respectively. Accompanying the decline in hepatic LA and ALA was an increase in hepatic mead acid (red arrow, ◄) **Fig 11D & 11E)**. Mead acid is a well-established marker of essential fatty acid deficiency (EFAD) [63]. Thus, consumption of the WD, which is low in EFAs (**S1A Fig**) is sufficient to decrease hepatic LA and ALA and increase MA, the EFAD marker.

We next examined the effect of the WD hepatic content LA, ALA and palmitic acid (PA, C16:0) and their elongation, desaturation and peroxisomal β-oxidation (pβOX) products (**Fig 12**). These results are expressed as fatty acid μmoles/g hepatic protein (Mean ± SEM) to provide insight into the hepatic abundance of these fatty acids. Hepatic LA content decreased significantly in response to feeding the EFA-deficient WD and this decline was rapid, ~50% reduction after 1 wk on the WD (**Fig 12, panel A**). Surprisingly, arachidonic acid (ARA, C20:4, ω6), a desaturation and elongation product of LA, changed little in response to the WD in both female and male mice. The end product of the ω6 PUFA pathway is the C_22_ ω6 PUFA, docosapentaenoic acid (DPA, 22:5, ω6); and ω6 DPA increased ~3-fold in livers of female mice fed the WD for 1 wk. Hepatic ω6 DPA remained elevated throughout the 40 wk WD feeding period in female mice. In male mice, ω6 DPA was lower in both LFD and WD fed mice and hepatic levels of this fatty acid were not responsive to the WD.

The relative abundance of LA: ARA: ω6 DPA in livers of female mice fed the LFD was 400:35:2.5 μmoles/g hepatic protein, respectively. Hepatic ω6 DPA is a minor hepatic fatty acid, its hepatic abundance is affected not only by diet, but also by sex. While LA and ARA are substrates for the synthesis of bioactive oxylipins, some investigators suggest ω6 DPA may improve blood lipids, i.e., LDL-C and HDL-C [64] as well as neural inflammation in an APOE-based Alzheimer’s disease model [65].

The level of ALA in the LFD and WD is 5.14 and 0.45 mole %, respectively (**S1A Fig**). The mole ratio of ω6/ω3 PUFA in the LFD and WD is 9.25 and 11.43, respectively. The decline in hepatic ALA in response to WD feeding parallels the decline in hepatic LA (**Fig 12A & 12B**). Fatty acid desaturation (Fads1, Fads2), elongation (Elovl2, Elovl5) and peroxisomal peroxidation (pβOX) converts ALA to EPA and DHA (**Fig 12 panel B**) [66]. Hepatic EPA and DHA levels fall paralleling the decline in ALA. The overall decline in ALA, EPA and DHA after 8 wks on the WD was ~90%, ~80%, ~50%, respectively. In contrast to LA, ALA elongation, desaturation and peroxisomal β-oxidation to form C_20-22_ PUFA was not affected by sex; both female and male mice had similar profiles of ALA, EPA and DHA in response to the WD.

Since EFAs in the WD are low and we identified a significant effect of the WD on hepatic pathways for LA and ALA conversion to C_20-22_ ω3 and ω6 PUFA, we examined WD effects on hepatic mead acid, an EFAD marker. Accordingly, the effect of the WD on hepatic palmitic acid (PA, 16:0), oleic acid (OA, 18:1, ω9) and MA (20:3, ω9) content over the time course of WD feeding is illustrated in **Fig 12C**. The accumulation of PA and OA in liver is due to both dietary sources (**S1A Fig**) and *de novo* lipogenesis (DNL), desaturation (stearoyl CoA desaturase, Scd1) and elongation (Elvol5 and/or Elovl6) to form OA [66,67]. Further elongation (Elovl5) and desaturation (Fads1, Fads2) converts OA to MA [63]. The pattern of change in PA and OA essentially paralleled the changes in MAS (**Fig 6**). Note that PA and OA in livers of female mice fed the LFD for 1 and 40 wks is 538 ± 19.4 and 600 ± 10.8 μmoles/g hepatic protein, respectively. MA is a low abundance hepatic fatty acid at 0.13 ± 0.01 and 0.06 ± 0.018 μmoles/g hepatic protein in LFD-fed female and male, respectively. MA is lower in livers of male mice when compared to female mice. In female mice, hepatic MA content increased to 0.82 ± 0.08 μmoles/g hepatic protein after 4 wks on the WD and stayed elevated throughout the duration of the feeding study. In male mice MA increased to 0.29 ± 0.015 μmoles/g hepatic protein after 4 wks of WD feeding. These results reveal a sex and diet effect on hepatic EFA and MA content. The elevation of MA in response to the WD was associated with the onset of insulin resistance and the progression of NAFLD to NASH (**Figs 4 and 9**). Clearly, low dietary EFA content in the WD induces hepatic levels of the EFAD marker, mead acid.

Analysis of plasma fatty acyls revealed low levels of both ω3 and ω6 PUFA in WD-fed mice, but not age-matched the LFD-fed mice [14]. We and others have reported that high fat diets like the WD lower the hepatic abundance of essential fatty acids [EFAs: LA & ALA [37,38,42–44]. Moreover, several clinical studies report that as NAFLD severity progresses, i.e., NAFLD transition to NASH and cirrhosis, hepatic EFAs and their products decrease [37–40,42–45,47]. A recent report indicates that key ω6 and ω3 C_18-22_ PUFA are low, while mead acid (20:3, ω9) is elevated in plasma of humans with biopsy-confirmed NASH [68]. Explanations for these observations are based on the low levels or an imbalance of EFAs in western-like diets [26,69] (**S1A Fig)** and the impairment in hepatic desaturation and/or elongation pathways required to convert the C_18_ EFA precursors to C_20-22_ ω3 and ω6 PUFA products [38,70]. A surprising outcome of our study was the major difference in how the WD affected MA content. Females have a more robust response than males to the WD in terms of the increase in hepatic MA in WD-fed mice. Differences in ω3 PUFA metabolism in male and female rodents have been reported previously [71].

### WD affects hepatic ω6 and ω3 PUFA-derived oxylipins

The decline in hepatic LA and ALA and their desaturation and elongation products described above prompted an analysis of hepatic oxylipins derived from ω3 and ω6 PUFA. We previously reported that feeding male and female *Ldlr*^*-/-*^ mice the WD not only affects hepatic SFA, MUFA and PUFA composition, but also significantly alters hepatic oxylipin type and abundance [43,47]. Enzymatically generated oxylipins have the potential to regulate multiple hepatic functions relevant to NASH onset and progression, such as inflammation, vascular compliance (vasoconstriction and relaxation), cell signaling, innate immunity and hepatocyte metabolism through plasma membrane and nuclear receptors [72–75]. However, these regulatory oxylipins are evanescent; they are short-lived while the inactive metabolites have a longer half-life and are more readily detected. Accordingly, we assessed the effect of the WD on LA, ARA, EPA and DHA-derived oxylipins (**Figs 13 & 14**).

The analysis focused on the hepatic non-esterified fatty acid precursors and oxylipins, since precursor fatty acids are enzymatically excised from membrane lipids by phospholipases and serve as substrates for enzymatic conversion to oxylipins [72,73]. In addition, we also quantified three non-enzymatically-derived oxylipins, i.e., 8-iso-PGF2α (**Fig 13**) derived from ARA and 18-HEPE & 8-iso-PGF3α derived from EPA. The arrows (◄) in **Figs 13 & 14** point to non-enzyme-generated oxylipins. These oxylipins form in membranes as a result of non-enzymatic lipid peroxidation and are excised from membranes by phospholipases and appear in the non-esterified fatty acid fraction of cells. As such they are markers of oxidative stress.

During the course of these studies, we discovered that the hepatic abundance of several oxylipins differ significantly in the LFD groups fed for 1 versus 40 wks **(Figs 13 & 14**). We attribute these differences to aging. As such, our analysis will focus on those oxylipins that are not significantly affected by aging in the 1 and 40 w LFD groups.

### ω6 PUFA-derived oxylipins

WD effects on hepatic non-esterified LA (18:2, ω6) and ARA (20:4, ω6), as well as non-esterified oxylipins derived from LA and ARA are illustrated in **Fig 13**. We quantified hepatic non-esterified LA and 6 oxylipins derived from LA. Hepatic levels of non-esterified LA and LA-derived oxylipins were not significantly by the WD after 1 wk, but were significantly lower after 40 wks on the WD in female and male mice. The suppressive effects of the WD on these fatty acids were apparent after 4 wks on the WD. The affected LA derived oxylipins include: 9(S)-HODE, 13(S)-HODE, 9,10-EpOME, 9,10-DiHOME and 12,13-DiHOME). Changes in hepatic levels of these oxylipins paralleled the decline in non-esterified LA. The WD suppressed hepatic 9(S)-HODE abundance by 40 to 60% after 4 wks on the WD and this effect persisted to 40 wks on the WD in both female and male mice **(Fig 13C)**.

9(S)-HODE and 13(S)-HODE are bioactive lipoxygenase products, while 9,10- and 12,13-EpOME are bioactive epoxygenase (Cyp2c) products [72–77]. These oxylipins are ligands for PPAR nuclear receptors and plasma membrane G-protein receptors. Ligand mediated receptor activation alters chemokine and interleukin (IL1β) production as well as neutrophil respiratory burst, mitochondrial function and cell survival. The epoxy-fatty acids (9,10-EpOME and 12,13-EpOME) are converted to inactive dihydroxy fatty acids (DiHOME) by a soluble epoxide hydrolase (Ephx2).

Circulating levels of oxidized LA metabolites, like 9(S)-HODE have been reported to impair mitochondrial function and promote apoptosis and NLRP3 activation [78]. In contrast, our study shows that NAFLD progression to NASH was associated with a decline in hepatic LA-derived oxylipins. The results reported in **Fig 13** confirmed our previous studies using female and male mice [43,47].

While total hepatic ARA (20:4, ω6) levels were not significantly affected by the WD (**Fig 12A**), non-esterified ARA showed considerable variability in hepatic abundance over the 40 wk feeding period in both female and male mice (**Fig 13**). Twenty-two oxylipins derived from ARA were quantified. Nine oxylipins are products of the prostaglandin pathway, either as primary products, like PGE2 or as inactive metabolites, like 6-keto-PGF1α, an oxylipin derived from the prostacyclin, PGI_2_. The graph for PGE2 (**Fig 13D**) reveals a major decrease in hepatic PGE2 after feeding female and male mice the WD for 4 wks and this WD-mediated suppression persisted for the duration of WD feeding. Other prostaglandins and thromboxane B2 did not reveal a differential response to the WD versus LFD.

Hepatic leukotriene B4, a lipoxygenase product, decreased in hepatic content after 1 wk on the WD and this effect persisted to 20 and 40 wks in female and male mice, respectively. Leukotriene B4 regulates multiple leukocyte functions, such as aggregation, release of lysosomal enzymes and nitric oxide production. Other lipoxygenase products include the hydroxy-eicosatrienoic acids (HETE). Hepatic levels of several HETEs were affected by the WD, including 5(S)-HETE, (8(R)-HETE, 8(S)-HETE, 12(S)-HETE & 15(S)-HETE). The graph for 5(S)-HETE in **Fig 13E** reveals the effect of the WD on this oxylipin. The WD increased 5(S)-HETE ~2-fold after 1 wk on the WD. In female mice, the response of 5(S)HETE to the WD was robust, but biphasic, whereas in male mice the WD response was sustained for the 40 wk feeding period. Other HETEs, e.g., 8(S)-HETE showed opposite responses to the WD in female and male mice suggesting the influence of sex on the hepatic regulation of HETEs. The epoxy-eicosatrienoic acids (EET) are Cyp2C products, while the dihydroxy-eicosatrienoic acids (DiHET) are derived from the EET by the soluble epoxide hydrolase, Ephx2. There was no consistent response of these oxylipins to the WD in either female or male mice. Similarity, there was no effect of the WD on the lipid peroxidation marker, i.e., isoprostane, 8-iso PGF2α (see the red arrow (◄) in **Fig 13**).

The outcome of the ω6 PUFA-derived oxylipin analysis revealed major effects of the WD on the generation of LA-derived lipoxygenase and epoxygenase products. The WD effect on these products paralleled WD effects on hepatic total and non-esterified LA content (**Figs 12 and 13**). WD effects on ARA-derived oxylipins, however, were less robust (**Figs 12 & 13**).

*ω3 PUFA-Derived Oxylipins*. Analysis of WD effects on hepatic oxylipins derived from EPA and DHA are reported in **Fig 14**. The heat maps include non-esterified EPA (C20:5, ω3) and DHA (C22:6, ω3) as well as twelve oxylipins derived from EPA and 14 oxylipins derived from DHA. Most of the EPA- and DHA-derived oxylipins were products of epoxygenases (Cyp2C and Cyp2J) and an epoxide hydroxylase (Ephx2). Leukotriene B5 is a lipoxygenase product and is implicated in immune modulation [74]. 18-HEPE and 8-iso PGF3α are lipid peroxidation products; see (◄) in the heat maps. As mention in the introduction to the analysis of hepatic oxylipins, there appears to be a significant aging effect in both female and male mice on the hepatic abundance of ω3 PUFA-derived oxylipins. As such, our analysis will focus on oxylipins that have similar levels of hepatic abundance in mice fed the LFD for 1 and 40 wks.

Non-esterified EPA was significantly increased in both female and male mice after 1 wk on the WD. Since there is little ALA and no EPA in the WD, we suggest this increase is due to the induction of membrane remodeling and the release of EPA form membrane lipids. The only EPA-derived oxylipin that changed after 1 wk on the WD in was the non-enzymatically derived 18-HEPE. This rapid effect was evident in female, but not male mice. After 40 wks on the WD, EPA, 17,18-DiHETE, 18 HEPE decreased in livers of female mice. While 18-HEPE is reported to be a precursor of pro-resolvin (RvE 1–3) [79], we detected no significant effect of the WD on hepatic RVEe1 abundance in female or male mice. In male mice, EPA and leukotriene B5 decreased while 14,15 DiHETE and the non-enzymatically generated lipid oxidation product, 8-iso PGF3α (◄) increased. Overall, these results indicate that hepatic non-esterified EPA and multiple Cyp2C/Ephx2 products decreased significantly in female and, male after 4 wks on the WD. Moreover, there was also a decline in many of these oxylipins after 40 wks on the LFD, making interpretation of these effects challenging.

DHA is the predominant ω3 PUFA accumulating in liver [43–45,47]. Of the 16 DHA-derived oxylipins examined, 9 and 8 DHA-derived oxylipins were significantly affected by the WD in female and male mice, respectively (**Fig 14**). In contrast to the EPA-derived oxylipins, many of the DHA-derived oxylipins in female and male mice were less affected by aging. We illustrate the effect of the WD on the following DHA-derived oxylipins, 10,11-EpDPA, 19,20-EpDPA and 10,20-DiHDPA in **Fig 14C and 14D**. The hepatic level of the epoxy- and dihydroxy-oxylipins was decreased after 4 wks on the WD. Also, no DHA-derived resolvin or protectin was significantly affected by the WD. Overall, the decline in most DHA-derived oxylipins paralleled the decline in non-esterified DHA.

The key outcomes of this analysis are that hepatic levels LA- and DHA-derived oxylipins are suppressed after 4 wks on the WD and this suppression was sustained for 40 wks while on the WD. There is also evidence of aging effects on hepatic levels of oxylipins since levels of several oxylipins from mice fed the LFD for 40 wks were lower than oxylipins after 1 wk on the LFD. The WD-regulated LA-derived oxylipins include epoxygenase, epoxide hydrolase and lipoxygenase products, while the DHA-derived oxylipins are epoxygenase and epoxide hydrolase products. While several oxylipins have the potential to play a regulatory role in hepatic function, the changes described above are not consistent with a role in controlling plasma TNFα (**Fig 4**) or the hepatic inflammation score (**Fig 9**); both increased significantly after 1 wk on the WD. Instead, changes in LA- and DHA-derived oxylipins may impact later events, such as the accumulation of lipid (steatosis) as well as fibrosis that appear in livers after 8–20 wks on the WD (**Figs 5, 8 & 9**).

### WD effects on hepatic gene expression

To gain insight into potential mechanisms controlling hepatic lipid levels and various markers associated with NASH, we examined WD effects on hepatic gene expression. The transcripts examined by qRTPCR in this report were based on transcripts quantified in our previous studies [43,47]. **S1 Table** contains DNA sequence data for all primers used for the qRTPCR analysis reported herein. The reference gene for the qRTPCR was cyclophilin. Results in heat maps are the mean value for mRNA abundance Delta C_T_, while results presented in graphs are the mean Delta CT value ± SEM.

### Expression of proteins involved in hepatic lipid metabolism

We first examined diet effects on hepatic expression of genes involved in lipid metabolism. Of the 33 transcripts examined (**Fig 15**), 19 and 15 transcripts were significantly affected by the WD in female and male mice, respectively. Long chain acyl CoA synthetase-long (Acsl) plays a key role in the synthesis of fatty acyl CoAs. In livers of female mice, the abundance of transcripts encoding Acsl1, 3 and 4 decreased rapidly (within 1 wk) in response to the WD. In male mice, the WD-mediated decline in acyl CoA synthetase-long chain (Acsl) mRNA abundance was apparent after 4 wks on the WD. Lysophosphatidylcholine acyl transferase (LpCat) subtypes transfer fatty acyl chains to lysophospholipids and likely play a role in membrane remodeling. All three LpCat subtypes (LpCat1, LpCat2 and LpCat4) were expressed at low levels in livers of LFD fed mice and feeding mice the WD increased hepatic mRNA abundance of all 3 LpCats in both female and male mice. The WD effect on LpCat2 (◄) is illustrated in **Fig 15C**. The hepatic abundance of LpCat2 mRNA trended upward after 1 wk on the WD and showed a progressive increase over the 40 wks on the WD. After 40 wks on the WD, LpCat2 was induced 4.2- and 2.6-fold in female and male mice, respectively.

In contrast to the LpCats, expression of peroxisome proliferator activated receptor (PPAR) target genes, i.e., acyl CoA thioesterase-1 (Acot1), Cyp4A10 and acyl CoA oxidase (Aox) declined after 4 wks on the WD and stayed low for the duration of the 40 wk WD feeding study. PPARs are regulated by both fatty acids and oxylipins [66,72]. As such, the WD-mediated decline in hepatic C_18-22_ ω3 and ω6 PUFA and oxylipins derived from these fatty acids (**Figs 11–14**) may account for the decline in expression of these transcripts. The effect of the WD on a representative PPAR target gene (Aox, ◄) is illustrated in **Fig 15B**. Hepatic Aox mRNA abundance trended downward as early as 1 wk on the WD and showed a significant (> 60%) decrease when compared to the LFD group after 40 wks on the WD. Hepatic abundance of transcripts encoding PPARα, PPARβ, PPARγ1, PPARγ2 and PPARγ co-activators (Pgc1α andPgc1β) were affected by diet, but the pattern of WD-induced change did not parallel the changes in Acot1, Cyp4A10 or AOX. This outcome suggests that changes in hepatic fatty acids and/or oxylipins levels, rather than changes in PPAR/co-activator expression, may account for the changes in hepatic mRNA abundance of the PPAR-target genes.

Sterol regulatory element binding protein-1c (Srebp1c) is a key transcription factor involved in regulating the expression of multiple transcripts encoding proteins involved in *de novo* lipogenesis [fatty acid synthase (Fasn), fatty acid desaturation, (Fads1, Scd1) and fatty acid elongation (Elovl3)] [80,81]. The mRNAs encoding these proteins increased in parallel after 4 wks of WD feeding. A representative transcript for this group (Scd1, ◄) is plotted in **Fig 15C**. After 4 wks on the WD hepatic Scd1 mRNA was increased 6- to 8-fold in livers of female and male mice and this transcript remained high for 40 wks on the WD. However, Scd1 mRNA increased in LFD-fed male mice after 40 wks revealing an aging effect on Scd1 expression. The other Srebp1c responsive genes (Fasn, Elovl3) were also induced in response to the WD. The time frame for the induction of these transcripts, i.e., from 4 to 40 wks of WD feeding, parallels the time course for WD-induced insulin resistance (**Fig 3C**) and the decrease in hepatic C_18-22_ ω3 PUFA (**Fig 11**). Srebp1c is a well-established target for fatty acid regulation. C_20-22_ ω3 PUFA suppress Srebp1 expression by affecting the transcription of the Srebp1 gene, Srebp1 mRNA stability and Srebp1c nuclear protein content [66]. The WD-mediated depletion of hepatic C_18-22_ ω3 and ω6 PUFA is consistent with the induction of Srebp1c expression, as well as the expression of its target genes (Fasn, Scl1, Elovl3).

Diacylglycerol acyl transferases (Dgat1, Dgat2) are key enzymes involved in the terminal stage of TAG synthesis. Hepatic Dgat1 mRNA abundance was rapidly decreased in female mice, but slowly decreased in male mice, in response to the WD. Dgat2 mRNA abundance was not affected by the WD in female mice, but was significantly decreased by the WD after 1 wk in male mice. Hepatic mRNA abundance of two TAG hydrolases (Tgh, Ces1g) decreased in response to the WD and were also affected by aging. While the mRNA encoding perlipin 2 (Plin2), a key lipid droplet protein, was suppressed rapidly (within 1 wk) by the WD in livers of female mice; Plin2 mRNA abundance was significantly suppressed after 40 wks. In male mice, Plin2 was significantly decreased after 40 wks on the WD.

Hydroxymethylglutaryl-CoA reductase (HmgCoA Red) and hydroxymethylglutaryl-CoA synthase 1 (HmgCoA Syn1) are key enzymes involved in cholesterol synthesis. Hepatic abundance of these mRNAs was rapidly reduced by the WD in female mice. Expression of these enzymes was not significantly affected by the WD in male mice. Sterol-O-acyltransferases (Soat1, Soat2) are involved in cholesterol ester synthesis. Soat1 mRNA abundance was significantly different after 1 wk on the WD in male mice, while Soat2 mRNA abundance was significantly different after 40 wks in female mice. Finally, Cyp7A1 is a key enzyme involved bile acid synthesis. Its mRNA abundance was not significantly affected by the WD in female and male mice.

The outcome of this analysis revealed significant effects of the WD on the expression of multiple genes involved in membrane lipid remodeling (LpCat1, LpDat3, LpCat4), PPAR signaling (Acot1, Aox & Cyp4A10 and Srebp1 signaling (Srebp1, Fasn, Scd1). Some transcripts do not respond similarly to the WD in female and male mice, e.g., HmgCoARed, HmgCoASyn1, Soat1 and Soat2. The lack of consistent effects of WD in male and female mice suggests that changes in the expression of these genes may not play a major role in WD-induced liver pathology.

### Expression of enzymes involved in oxylipin metabolism

We previously reported that the WD consistently affected the hepatic expression of prostaglandin synthease-2 (Ptgs2), but had little effect on other enzymes involved in hepatic oxylipin metabolism [43,47]. Herein, we re-examined the effect of the WD on the expression of these enzymes to determine if sex and duration of WD feeding affected their expression (**Fig 16**).

The WD significantly induced Ptgs1 expression (~2-fold) in both female and male mice after 1 wk. In female mice, but not male mice, Ptgs1 mRNA abundance increased progressively over the 40 wks feeding period. Ptgs2 mRNA was significantly increased (~4-fold) after 1 wk of WD feeding in female, but not male mice. However, Ptgs2 mRNA abundance increased progressively in both female and male mice over the 40 wk WD feeding period. Ptgs1 and Ptgs2 are not expressed in hepatocytes, but expressed in other hepatic cells, including cholangiocytes, endothelial, stellate and Kupffer cells as well neutrophils (https://www.proteinatlas.org/. The rapid effects of the WD on Ptgs1 and Ptgs2 expression suggests that cells expressing these enzymes are early targets of WD effects on the liver. Despite the rapid effects of WD on Ptgs1 and 2 mRNA abundance, rapid and sustained changes in hepatic Pge2 were not observed (**Fig 13**). Instead, the hepatic abundance of the inactive prostaglandin metabolite, e.g., 15-keto PGE2 (**Fig 13, ◄**) increased after 4 wks on the WD. This finding suggests WD-induced PGE2 synthesis, but also the rapid conversion of PGE2 to an inactive metabolite, 15-keto-PGE2.

The hepatic abundance of mRNAs encoding epoxygenases (Cyp2c and Cyp2J5) and epoxide hydrolases (Ephx1 and Ephx2) were also affected by the WD, but the response to the WD did not reveal a consistent and significant change over time. The overall outcome of the oxylipin analysis suggests that the WD significantly affects the hepatic abundance of oxylipins derived from LA, ARA (**Fig 13**) and DHA (**Fig 14**) after 1 wk on the WD. This timeline for the WD-induced change in oxylipins coupled with the rapid effects on Ptgs1 and Ptgs2 expression, suggests that LA-, ARA- and DHA-derived oxylipins may be involved in the early regulation of systemic and hepatic inflammation after 1 wk on the WD (**Figs 4 and 9**).

### Expression of proteins involved in hepatic inflammation

Of the 28 transcripts associated with hepatic inflammation examined in **Figs 17 and 18**, 19 and 22 transcripts were significantly affected by the WD in female and male mice, respectively. Data for several of these transcripts, designated by an arrow in the heat maps (◄), was graphed in **Fig 18A–18H** to illustrate the effect of sex and time on the response of these transcripts to the WD.

Monocyte chemoattractant protein-1 (Mcp1) is expressed in hepatic cholangiocytes, endothelial cells and neutrophils (https://www.proteinatlas.org/) and this gene is an early responder to factors promoting inflammation. Hepatic expression of Mcp1 is low in LFD fed mice, but the WD increased Mcp1 mRNA significantly after 1 wk (**Fig 18A**). After 40 wks on the WD, Mcp1 was induced 18-fold in female and male mice. In contrast to male mice, female mice have a more robust induction of Mcp1 after 1, 4 and 8 wks on the WD. CD40 is a member of the TNF receptor super gene family and is expressed in multiple hepatic cells. Hepatic CD40 is expressed at levels lower in male mice than in female mice. CD40 mRNA increased in livers of female and male mice by ~50% after 1 wk on the WD (**Fig 18B**). Overall, CD40 mRNA increased 3-fold after 40 wks on the WD. CD68 is a macrophage marker and its expression was higher in LFD-fed female mice than male mice (**Fig 18C**). After 40 wks on the WD, CD68 was induced 5.7- and 3.1-fold in female and male mice, respectively. F4/80 is a G-protein receptor and a macrophage marker. Its expression in LFD-fed mice was lower in male mice than female mice (**Fig 18D**). F4/80 mRNA was significantly induced in male, but not female mice after 1 wk on the WD. After 40 wks on the WD, F4/80 mRNA levels were increased 3.7- and 2.2-fold in female and male mice respectively. NLR family pyrin domain containing 3 (Nlrp3) is a key protein involved in inflammasome organization and is expressed in hepatic cholangiocytes, endothelial and Kupffer cells and neutrophils [82]. Expression of Nlrp3 was rapidly induced (2.4-fold) by the WD within 1 wk on the WD feeding in female but not male mice (**Fig 18E**). The overall induction of Nlrp3 after 40 wks on the WD was 2.5-fold in both female and male mice. Tumor necrosis factor α (Tnfα) plays a major role in systemic and hepatic inflammation and in the progression of NAFLD to NASH [83]. In liver, Tnfα is expressed in cholangiocytes, Kupffer and T-cells. Hepatic expression of Tnfα was rapidly induced (3.7-fold) in livers of female, but not male mice after 1 wk on the WD (**Fig 18F**). After 40 wks on the WD, Tnfα mRNA was increased 5.8- and 3.3-fold in female and male mice, respectively. Glycoprotein nmb (Gpnmb) is another macrophage product, but also expressed in multiple hepatic cells. Gpnmb is a blood biomarker for NAFLD; and some suggests Gpnmb may have beneficial effects on NAFLD [84,85]. Gpnmb expression in livers of LFD fed female and male mice was very low. Hepatic abundance of Gpnmb rapidly increased ~7-fold in female, but not male mice in response to 1 wk of WD feeding (**Fig 18G**). The mRNA encoding Gpnmb in female and male mice was induced 32- and 17-fold, respectively, after 40 wks on the WD. Finally, the triggering receptor expressed on myeloid cells (Trem2), like Gpnmb, is another circulating marker of NAFLD. Trem2 plays a protective role in liver by modulating hepatic TLR4 action [86–88]. In LFD-fed mice hepatic Trem2 is expressed at low levels. After 1 wk on the WD, Trem2 was induced 4.2- and 2.1-fold in livers of female and male mice, respectively. After 40 wks on the WD, Trem2 mRNA was increased 12.4- and 8.6-fold in female and male mice, respectively.

The outcome of this analysis has established that 1 wk of WD feeding was sufficient to significantly induced multiple mRNAs linked to hepatic inflammation (**Figs 17 & 18)**. With the exception of F4/80, levels of transcript induction were greater in female mice than male mice. Moreover, after 40 wks on the WD, female mice had higher levels of transcript abundance than male mice. These results indicate that female mice have a more rapid and robust response to WD feeding than male mice. More important, the onset of change in expression of many of these transcripts precedes any evidence of obesity, insulin resistance, histological evidence of NASH, i.e., steatosis and fibrosis. Instead, the change in expression of these transcripts is inversely associated with the timeline for the WD-mediated decline in hepatic EFAs (**Figs 11–13**).

### WD effects on oxidative stress, apoptosis, fibrosis, extracellular matrix remodeling and cell growth

#### Oxidative stress

Oxidative stress plays a key role in the onset and progression NAFLD to NASH, cirrhosis and HCC [89–91]. We quantified transcripts encoding three proteins linked to cellular oxidative stress (**Fig 19A & 19B**), including cytochrome B-245 β chain (Cybβ) a component of the NADPH oxidase complex, glutathione S-transferase α1 (Gstα1), and heme oxygenase 1 (Hmox1). All three transcripts trended upward after 4 and 20 wks on the WD in female and male mice, respectively. We also quantified hepatic reduced (GSH) and oxidized glutathione (GSSG) and the GSH/GSSG ratio in the heat map and the **Fig 19A–19C**. Changes in the GSH/GSSG ratio reflect changes in oxidative stress status within cells where a low GSH/GSSG ratio indicates increased oxidative stress and/or decreased recycling of GSSG to GSH. As illustrated in **Fig 19C**, LFD-fed female and male mice have a low GSH/GSSG ratio at both 1 and 40 wks. This is expected since the mice were fasted overnight prior to euthanasia (Methods) and fasting is associated with a decline in tissue GSH levels [92]. One wk on the WD does not change this ratio, but afterward, this ratio revealed a biphasic response to the WD. The GSH/GSSG ratio was increased by 4 wks followed by a decline beginning after 20 wks on the WD. Interestingly, this change in the GSH/GSSG ratio paralleled the changes in Homa-IR and MAS (**Figs 3, 5 & 6)**. Finding GSH/GSSG values above values measured in LFD fed mice suggests that livers of WD-fed mice likely have sufficient capacity to maintain GSH to prevent robust oxidative stress damage. This finding may explain why we did not find increased formation of the lipid peroxidation markers, derived from LA or EPA, i.e., 8-iso PGE2 and 8-iso-PGE3 (**Figs 13 & 14**).

#### Apoptosis and autophagy

We next examined the expression of caspase 1 (Casp1) and two cathepsins (CtsB and CtsS). Casp1 is involved in apoptosis, while the cathepsins are lysosomal proteases involved in autophagy and the turnover of intra- and extra-cellular proteins. Both Casp1 and the cathepsins play a role in NASH pathology [93,94]. All 3 markers were significantly induced after 40 wks on the WD. We also plotted the qRTPCR data for CtsS (**Fig 19D**). Interestingly, CtsS expression was inversely associated with the hepatic GSH:GSSG ratio. GtsS expression is highest when the hepatic GSH:GSSG ratio is low. This result may reflect a role of oxidative stress in the regulation of CtsS expression.

### Fibrosis and Extracellular Matrix Remodeling (ECMR)

Hepatic fibrosis is the hallmark of significant hepatic injury; and injury promoted scarring involves extracellular matrix remodeling (ECMR). As shown in the heat maps (**Fig 19**) and graphs (**Fig 20A–20D**) collagen 1A1 (Col1A1), lysyl oxidase (LysOx), matrix metalloproteases (Mmp12, Mmp13) and tissue inhibitor of metalloprotease (Timp1) are well induced after 40 wks on the WD. A closer look at the heat maps suggest that the expression of some of the fibrosis and ECMR markers were affected by the WD after 1 wk on the WD. Moreover, there is a trend for a more rapid increase in these markers in response to the WD in female mice than male mice. Accordingly, we assessed these differences in **Fig 20A–20D,** where we graphed the qRTPCR data for Col1A1, Tgfβ1, Mmp12 and Timp1. Col1A1 is expressed in hepatic stellate cells. In whole liver, Col1A1 is expressed at low levels in LFD-fed mice. Its mRNA increased ~2-fold after 1 wk on the WD in female and male mice. The overall effect of the WD on Col1A1 abundance between female and male mice at 40 wks on the WD versus LFD was 19.6- and 6.5-fold, respectively. The elevated expression of Col1A1 at 20 and 40 wks of WD feeding correlated with the appearance of PSR staining for fibrosis (**Figs 8 & 9**). Tgfβ1 mRNA displayed a different response to the WD. Tgfβ subtypes are major regulators of hepatic Col1A1 expression and fibrosis [95,96]. Tgfβ1 is expressed in multiple hepatic cells, but not hepatocytes. The transcript encoding Tgfβ1 was induced 2-fold in female, but not in male mice after 1 wk on the WD. After 40 wks on the WD, Tgfβ1 mRNA was increased 2.7- and 2.1-fold in female mice and male mice, respectively.

The induction of hepatic fibrosis is associated with an increase in expression of enzymes involved in ECMR. We examined the effect of the WD on two transcripts encoding proteins involved in ECMR, i.e., matrix metalloprotease-12 (Mmp12) and tissue inhibitor of metalloproteases-1 (Timp1). Like Col1A1, hepatic expression of Mmp12 and Timp1 is very low in livers of LFD-fed mice (**Fig 10C & 10D**). Mmp12 was induced 4- and 2.3-fold after 1 wk on the WD in female and male mice, respectively. After 40 wks on the WD, Mmp12 was induced 73- and 25-fold in female and male mice, respectively. Timp1 was induced 2.5- and 1.8-fold in female and male mice after 1 wk on the WD and after 40 wks on the WD, Timp1 was induced 23 and 12-fold, respectively. The outcome of this analysis revealed an apparent coordinate induction of expression of proteins involved in fibrosis and ECMR over the 40 wk WD feeding study. These results also reveal significant changes in hepatic expression of Col1A1, Mmp12 and Timp1 well before the appearance of histological evidence of hepatic fibrosis (**Fig 8**).

### Hepatic cell growth and differentiation

NASH can progress to cirrhosis, hepatocellular carcinoma and liver failure. This transition is associated with significant changes in hepatic cell survival, cell type and cell signaling through the activation of pathways like notch and hedgehog signaling [97,98]. We previously reported that several gene expression markers linked to these pathways were regulated by the WD in our *Ldlr*^*-/-*^ mouse model [43]. The heat map and graphs **(Figs 19 & 20E–20I)** describe the time course for WD induced changes in hepatic expression of the following proteins in livers of male and female mice; including: 1) B-cell lymphoma 2 (Bcl2), 2) growth arrest and DNA damage (Gadd45), 3) Notch signaling proteins Hey1, HeyL, 4) Indian hedgehog (Ihh) and hedgehog interacting protein (Hhip), 5) SRY-box containing gene 4 (Sox4), a transcription factor and 5) the transcriptional repressor (Snai1/Snail1).

While the WD significantly affected the hepatic expression of most of these markers (**S1C and S2 Figs**), only Bcl2, Hey1, HeyL, Hhip and Sox4 were significantly changed after 1 and/or 40 wks in female and/or male mice fed the WD. To understand how these proteins function in liver biology, we used the human protein atlas (https://www.proteinatlas.org/) to determine which hepatic cells expressed these proteins. Accordingly, hepatic Bcl2 is expressed in cholangiocytes (bile duct), vascular endothelial, stellate, Kupffer and T-cells. Bcl2 regulates apoptosis and cell survival by its effects on mitochondria [99]. Hey1 and HeyL are downstream mediators of Notch signaling. These transcription factors play key roles in vascular development, as well as extracellular matrix remodeling (ECMR) and fibrosis [100,101]. These proteins are expressed in hepatic endothelial cells, particularly cholangiocytes and vascular endothelial cells. In addition, Hey1 is expressed in hepatic neutrophils, while HeyL is expressed in hepatic stellate cells. The hedgehog markers, Indian hedgehog (Ihh) and hedgehog (Hh) interacting protein (Hhip), are expressed in hepatocytes and stellate cells, respectively [43]. Hedgehog (Hh) signaling is required for liver regeneration, regulation of capillarization, control of the fates of hepatic stellate cells, and promoting liver fibrosis and liver cancers [102]. Hhip interferes with the capacity of Hh ligands to mediate changes in cell function [103]. Sox4 is expressed in hepatic cholangiocytes and vascular endothelial cells. This protein plays a role in multiple cell development pathways, as well as triglyceride metabolism and liver steatosis [104,105].

Hepatic expression of Bcl2 was significantly increased in liver after 40 wks on the WD (**Fig 19**). In female and male mice, Bcl2 mRNA trended higher after 4 and 20 wks, respectively, on the WD (**Fig 20E**). While both Hey1 and HeyL were significantly affected by the WD after 40 wks on the WD (**Fig 19**), only HeyL was significantly increased after 1 wk on the WD in both female and male mice (**Fig 20F**). Ihh is a hedgehog signaling ligand and its mRNA abundance trended upward after 4 wks on the WD in male mice and 8 wks in female mice. Ihh was significantly increased after 40 wks on the WD in both female and male mice fed the WD (**Fig 20G**). Interestingly, hepatic hedgehog interacting protein (Hhip) displayed the opposite response to the WD. Hepatic Hhip mRNA abundance decreased after 4 and 20 wks on the WD in females and males, respectively. In both groups of mice there was a progressive decline in hepatic Hhip mRNA in response to the WD (**Fig 20H**). If cellular Ihh and Hhip protein abundance parallel changes in Ihh and Hhip mRNA, then the inhibitory capacity of Hhip on Hh signaling would be expected to decrease more rapidly in livers of female mice than male mice in response to WD feeding. In female and male mice, Sox4 mRNA trended upward after 4 wks and 20 wks on the WD in female and male mice, respectively (**Fig 20I**).

The outcome of this analysis suggests that activation notch signaling (HeyL) is an early event in WD induced NASH, while hedgehog (Ihh and Hhip) and Sox4 signaling are activated after 4–20 wks on the WD. Also, there is a different timeline for the WD regulation of Bcl2, HeyL, Ihh and Sox4 in female and male mice. As such, this analysis reveals a sex- and time-dependent effects of the WD on the regulation of these pathways. Most notable is the rapid effect of the WD on the induction of HeyL mRNA, an event that precedes the appearance of histological evidence of NAFLD and NASH (**Figs 5, 8 & 9**).

## Discussion

The goal of this study was to establish the time course for western diet (WD)-induced NASH in the preclinical *Ldlr*^*-/-*^ mouse model to assess changes in anthropometric, plasma and hepatic markers of NASH. The analysis included histology, lipid (cholesterol, triglyceride, the fatty acid and oxylipin profile)] and gene expression assessments. Specifically, our goal was to: 1) identify early markers of WD-induced disease; 2) determine which lipids might serve as biomarkers of NASH; and 3) determine if female and male mice responded similarly to the WD. Our study identified early changes in systemic and hepatic inflammation markers that preceded overt evidence of NAFLD, i.e., obesity, insulin resistance, hepatic steatosis and fibrosis in age-matched female and male *Ldlr*^*-/-*^ mice. Our study also established that female and male mice do not have identical responses to the WD. Female mice are more responsive to the WD than male mice (**Fig 10**).

This study was carried out concurrently with age-matched female and male mice. The advantages of this study design are: 1) the approach avoids confounding outcomes due to differences in food batch composition, animal age or animal batch from the supplier, personnel handling the animals or vivarium conditions; 2) the approach reveals both similarities and differences in how age-matched female and male mice responded to the WD; 3) the longitudinal design of the study provides insight into the early NADLD markers and the changes in these markers over time 3) the approach increases the power of the analysis by increasing the number of animals in the study and 4) increases confidence in the outcomes when common markers are found to be affected by the WD in both female and male mice.

The prevailing view of diet induced NAFLD and its progression to NASH involves the accumulation of lipid (cholesterol, triglycerides, diacylglycerides and ceramides) in liver that promotes hepatic inflammation leading to hepatic injury, inflammation, oxidative stress and fibrosis [4,11–14]. The excessive accumulation of hepatic lipid is postulated to be the 1^st^ hit is a multi-hit pathway leading to NASH [12,13]. Absent from this model are the antecedent events preceding the abnormal accumulation of hepatic lipid. As mentioned earlier, the NIDDK considers NAFLD to be a “silent disease” with no obvious disease indicators. NAFLD is typically diagnosed in patients with pre-existing diseases, such as obesity, type 2 diabetes, dyslipidemia and MetS. Our study fills this gap and presents an alternative view of the time course for WD-induced NAFLD and its progression to NASH.

We list 14 markers of NAFLD/NASH that were quantified in this study (**Fig 21**). Most markers changed in response to the WD prior to histological evidence of macrosteatosis (MAS), which was apparent after 20 and 8 wks on the WD in female and male mice, respectively. While some of these changes were small, they were the beginning of a long and progressive change over time. For example, histological evidence of fibrosis was first detected after 8 wks on the WD in both female and male mice (**Figs 5, 6, 8 & 9).** However, gene expression analysis revealed a small, but significant increase in hepatic mRNAs encoding hepatic proteins involved in fibrosis (Col1A1, Tgfβ1) and ECMR (Mmp12 and Timp1) after 1 wk on the WD (**Fig 20**). Plasma markers of dyslipidemia (plasma cholesterol), insulin resistance (Homa-IR), and systemic and hepatic inflammation (Tnfα) were significantly increased prior to hepatosteatosis (**Fig 3, 4 & 18F**) and obesity (**Fig 2B**). Hepatic cholesterol, the new villain in the NAFLD landscape [50,52] was elevated after 1 wk on the WD in female and male mice and significantly increased after 40 wks on the WD (**Fig 6**). Metabolic endotoxemia (plasma TLR4-Ag) was elevated in female and male mice after 1 to 4 wks on the WD feeding (**Fig 4B & 4C**). This finding indicates rapid changes in the gut-liver axis in response to the WD [17,18]. Metabolic endotoxemia, i.e., plasma TLR4 agonist [18] and elevated hepatic cholesterol are key factors in the onset and progression of NASH [51,52]. Additional markers changed in both male and female mice prior to the appearance of MAS (**Figs 5, 6 and** 9) including the onset of insulin resistance (Homa-IR, **Fig 3**), changes in hepatic oxidative stress status (GSH/GSSG) (**Fig 19**) and a significant difference in body weight between LFD and WD-fed mice at 8 wks and LW%BW at 20 wks (**Fig 2B & 2C**). Taken together, these findings identify multiple early markers of NASH that precede histological evidence of the NAFLD and NASH (**Fig 21**). The early plasma markers (Tnfα, TLR4-Ag) may serve as targets for early clinical assessment and therapeutic intervention.

Another outcome of our analysis was that female and male mice respond differently to the WD. This is particularly evident in the response of female and male mice after 1 wk on the WD. Female mice have a more robust inflammatory response to the WD when compared to male mice (**Table 1** and **Figs 18 & 21**). The relevance of this finding to human NASH, however, is unclear. Hormonal status is known to affect the onset of NAFLD in humans. Premenopausal women are less likely to develop NASH and fibrosis than children, men, and post-menopausal women, [106,107]. Nevertheless, this difference in response of female and male mice to the WD may be relevant in early diagnosis of NAFLD. The physiological basis for this differential response to the WD will require further investigation. In addition to measuring plasma TNFα, clinical assessments of plasma Gpnmb [84] and Trem2 [86] might also serve as early indicators of hepatic and/or systemic inflammation.

Our third goal was to determine whether there was a temporal link between the onset of NASH and changes in hepatic EFAs [linoleic acid (LA, 18:2, ω6 and α-linolenic acid (ALA, 18:3 ω3)], and their oxylipin metabolites. As indicated earlier, hepatic levels of ω3 and ω6 PUFA in human liver decrease as the severity of hepatosteatosis progresses to NASH and cirrhosis [31–39]. In humans, the decline in hepatic C_18-22_ ω3 and ω6 PUFA is likely due to effects on hepatic PUFA metabolism or changes in hepatic cellular composition [14,38]. In mice, however, the decline in hepatic C_18-22_ ω3 and ω6 PUFA in response to the WD is associated with low blood levels of C_18-22_ ω3 and ω6 PUFA reflecting EFA deficiency. EFA deficiency in the *Ldlr*^*-/-*^ mouse/WD preclinical model is due, at least in part, to the low level of LA and ALA in the WD (**S1A Fig**). Clearly, WD feeding promotes a rapid loss of hepatic EFAs with significant decline in LA and DHA-derived oxylipins (**Figs 11–13 & 21).** While one week on the WD was sufficient to significantly lower LA and ALA in livers of female and male mice (**Figs 11 & 12**), 4 weeks of WD feeding was required to observe a major decline in LA and DHA-derived oxylipins (**Figs 13 & 14**). The decline in hepatic LA- and DHA-derived oxylipins occurred as insulin resistance (Homa IR) and the GSH/GSSG ratio increased (**Figs 3, 13, 14 & 19**). Whether this observation is a cause & effect relationship or merely coincidence will require additional studies. Our data, however, clearly show that WD suppression of hepatic ω3 and ω6 PUFA and oxylipin content precedes WD-mediated induction of the major hepatic events linked to NASH, such as MAS and fibrosis.

In further support of the role EFA deficiency plays in WD NASH, we quantified hepatic levels of mead acid (C20:3, ω9), an EFA deficiency marker. The substrate for the synthesis of mead acid is oleic acid C18:1, ω9; and this fatty acid is elongated and desaturated to form 20:3, ω9 (**Fig 14C**). The accumulation of mead acid is likely due to three mechanisms. First, there is increased hepatic MUFA content arising from high MUFA content in the WD (**S1A Fig**, **Figs 11 & 12**). Second, the WD increases *de novo* lipogenesis increasing the production of palmitate and its subsequent elongation (Elovl3/Elovl5) and desaturation (Scd1) to form oleic acid (18:1, ω9) (**Fig 12C**) [108]. The third mechanism involves the lack of suppression of Scd1 gene transcription by DHA > EPA > LA. Scd1 was one of the first genes to be recognized as a target of C_20-22_ ω3 regulation of gene expression [109]. As such, the decline in hepatic DHA is associated with the increase in hepatic expression of Scd1. The mechanism for PUFA-mediated suppression of Scd1 expression involves suppression of Srebp1c nuclear abundance, a key transcription factor controlling expression of genes involved in *de novo* lipogenesis (DNL) and MUFA synthesis [66]. More recent studies have shown that adding C_20-22_ ω3 PUFA to the WD restores C_20-22_ ω3 PUFA in liver, as well as decreasing Scd1 expression and increasing oxylipins derived from C_20-22_ ω3 PUFA. Moreover, these changes in hepatic lipid are associated with attenuation of WD induced NASH as well as promotion of NASH remission, i.e., decreased hepatosteatosis [42,44,46]. Taken together with the clinical observations [33–35,37–39], these findings suggest that dietary EFA sufficiency is an important parameter in the clinical management of patients with NAFLD, from benign steatosis to HCC.

EFA deficiency or an imbalance of ω3 and ω6 PUFA, i.e., the ω6/ω3 PUFA ratio, received attention in a recently published longitudinal study [110]. The Raine study used a prospective cohort including 985 participants ranging in age from 14 to 22 years of age. Individuals at increased risk of developing fatty liver disease in early adolescence were associated with high dietary intake of ω6 PUFA and a high dietary ω6/ω3 PUFA ratio [110]. Children who developed NAFLD had increased risk of further complications in adulthood, like NASH, T2DM, cardiovascular and renal disease. Moreover, human clinical trials have established that efforts to lower the ω6/ω3 PUFA ratio through dietary supplementation with C_20-22_ ω3 PUFA decreased hepatosteatosis and liver injury [111–114]. This treatment strategy, however, did not reduce hepatic fibrosis [113,115,116].

Our findings, coupled with our previous studies using DHA to mitigate NASH onset and progression [42,44] support that notion that changes in hepatic C_18-20_ ω3 and ω6 PUFA play an important role in liver health. The significant decline in hepatic LA and ALA in response to 1 wk of WD feeding was inversely associated with significant increases in expression of multiple hepatic genes linked to inflammation (**Figs 11, 12, 16–18**). These hepatic gene expression markers include transcripts encoding Cd40, Cd44, Cd68, F4/80, Gpnmb, Mcp1, Nlrp3, Ptgs1, Ptgs 2, Tnfα, and Trem2 (**Figs 16 and 18A–18H**). These transcripts represent a subset of the transcripts that increased rapidly in livers in response to the WD and persist at elevated levels throughout the 40 wk WD feeding study. This raises the question of whether such changes in hepatic EFAs or their C_20-22_ oxylipin products directly affect hepatic abundance of inflammation markers. We addressed this issue in two previous studies, a NASH prevention [42,46] and a NASH treatment study [44]. Supplementing the WD with DHA attenuated the WD-mediated induction of these and other gene expression markers of inflammation [44,46] as well as reversing the effects of the WD on hepatic ω3 PUFA-derived oxylipins. These findings suggest that maintaining a level of C_20-22_ ω3 PUFA in the liver is critical to attenuate WD-induced hepatic inflammation and progression to NASH. We have established that induction of hepatic inflammation is an early event associated with WD induced NAFLD and this response precedes all other markers associated with NASH, such as obesity, insulin resistance, changes in hepatic GSH/GSSG ratio, and fibrosis (**Fig 21**).

Based on these findings, we recommend attention to dietary EFA intake as a potential target for therapy and the mitigation of NAFLD/NASH severity. The EFAs and their C_18-22_ oxylipin derivatives play multiple roles in cell function including: 1) serving as components of membrane lipids, 2) regulators of membrane lipid raft composition and signaling from membranes, and 3) conversion to oxylipins which are ligands for membrane and nuclear receptor signaling [66]. As we show in **Fig 11**, the mole% of ω3 and ω6 PUFA decrease while hepatic MUFA content increases. Such changes in hepatic fatty acid type is likely to lower membrane levels of ω3 and ω6 PUFA, which will influence membrane lipid composition and fluidity, signaling from the plasma membrane and the generation of regulatory oxylipins. Because of the pleiotropic role EFAs play in cell function, it is not surprising that a significant decline in their hepatic abundance affects multiple hepatic pathways. Hepatic oxylipins are transient, i.e., short term, regulators of multiple hepatic functions, including inflammation, blood flow and metabolism [78,117–120]. There are many hepatic oxylipins affected by the WD. As such, it will be challenging to assign changes in hepatic functions to specific oxylipins.

Preclinical and clinical studies report that dietary C_20-22_ ω3 PUFA supplementation of mice or patients with pre-existing NAFLD lowers hepatic fat and reduce hepatic injury [4,40,44,46,111,113,115]. However, both preclinical and clinical therapeutic studies reveal that dietary supplementation with C_20-22_ ω3 PUFA does not promote total removal of hepatic fibrosis [14]. A recent report indicates that the removal of excess ECM requires the matrix metalloprotease Mmp12 [121]. Mmp12 is expressed at low levels in liver, but is induced in response to the WD in livers of WD-fed mice (**Fig 20C**). While DHA supplementation of the WD lowers hepatic expression of Col1A1, Col1A2, it also lowers hepatic expression of several Mmps, including Mmp12. As such, DHA supplementation of the WD may block full removal fibrosis/ECM, as seen in liver histology [43,44]. This finding represents a significant limitation of C_20-22_ ω3 PUFA in NASH therapy. As such, EFAs and/or C_20-22_ ω3 PUFA should be considered for preventive use or use in patients who present with hepatosteatosis, but not fibrosis.

## Conclusions

Time is an important variable in dissecting cause and effect relationships in pathophysiology. Many studies on the effect of diet on the NAFLD and NASH use a single timepoint for their analysis. This report clearly shows that time is a critical factor in defining how dietary factors impact the onset and progression of NAFLD and NASH. Current NAFLD/NASH diagnoses and therapies are based on histological evidence of disease. Our findings indicate that changes in systemic and hepatic makers of inflammation occur rapidly after a dietary challenge using the western diet, and these events precede the development of histological evidence of MAS and fibrosis. Moreover, these silent events worsen over time to impact multiple regulatory pathways that impact the onset of MAS, liver injury, fibrosis and ECMR. Our studies establish that the increase in systemic and hepatic inflammation were inversely associated with a loss of hepatic EFA, implicating hepatic EFA status in maintaining liver health. While we understand how EFAs and their C_20-22_ ω3 and ω6 PUFA products regulate hepatic lipid metabolism [4,66], less clear is how EFA-derived oxylipins affect liver function. Several reports implicate a role for oxylipins in liver health [43,47,78,117–119], but the diversity of the hepatic oxylipin species and the rapid conversion of active oxylipins to inactive compounds complicates an assignment of cause and effect in the management of liver health. There are many dietary and environmental factors contributing to liver health and the onset of fatty and inflamed liver disorders [122]. In cases where NAFLD is attributed to poor diet, such as a diet with high ω6/ω3 PUFA ratio or low EFA content [23–29], we suggest correcting EFA status to aide in mitigating NASH severity.

## Supporting information

S1 Fig **A. Fig Diet Composition**. Composition of diets purchased from Research Diets: Low fat diet (LFD) and Western diet (WD). The fatty acid content of the diets is illustrated in the graph as Mole%. The Diets were extracted for lipid. The extracted lipid was saponified, methylated, fractionated and quantified by gas chromatography as described in the Materials and Methods. The Mole % of the essential fatty acids (18:2, ω6 and 18:3, ω3) and the mole % of 18:2, ω6 to 18:3, ω3 is presented in the figure. **B. Fig Histology of hepatosteatosis with inflammation.** Liver of a mouse fed the western diet for 40 wks was fixed, embedded, sliced and stained with hematoxylin and eosin as described in the Materials and methods. The histology slide was photographed at 400x and examined and labeled by a board-certified veterinary pathologist. **C. Fig Histology of hepatosteatosis: MIS & MAS.** Livers were prepared from mice fed the LFD (left panel) and WD (right panel) for histology as described in Materials and Methods. Liver slices were stained with hematoxylin and eosin and photographed at 400 X, as in S1B Fig. The liver samples were examined and labeled by a board-certified veterinary pathologist. **D. Fig Histology of hepatic fibrosis.** Liver of a mouse fed the western diet for 40 wks was fixed, embedded, sliced and stained with Picro sirius red as described in the Materials and methods. The histology slide was photographed at 40x (left) and 100x (right) and examined and labeled by a board-certified veterinary pathologist. **E. Fig Daily food consumption (A) and Cumulative calorie consumption (B).** Female and male mice were fed the LFD or WD for 40 wks. Mice were weight and fed twice weekly. [**A**] Food consumption was quantified twice weekly and expressed as Food consumption (grams/day/mouse) for both LFD and WD fed female and male mice twice weekly. [**B**] Cumulative calorie consumption was calculated by multiplying the daily food consumption (grams) by the caloric density of the LFD and WD, i.e., 3.82 and 4.67 kcals/g, respectively (see S1A Fig). Results are expressed as mean ± SEM.
(PPTX)

S1 Table qRTPCR primer data. This file contains the DNA sequence for each primer pair for each transcript quantified by qRTPCR reported in the manuscript.
(XLSX)

S2 Table (XLSX)

S3 Table (XLSX)

S4 Table (XLSX)
