# Supplementary material for: Time course of western diet (WD) induced nonalcoholic steatohepatitis (NASH) in female and male Ldlr-/- mice
Source: PLoS One. 2023 Oct 11;18(10):e0292432. doi: 10.1371/journal.pone.0292432 (PMC10566735; doi:10.1371/journal.pone.0292432)
Supplement: S1 Fig — A. Fig Diet Composition. Composition of diets purchased from Research Diets: Low fat diet (LFD) and Western diet (WD). The fatty acid content of the diets is illustrated in the graph as Mole%. The Diets were extracted for lipid. The extracted lipid was saponified, methylated, fractionated and quantified by gas chromatography as described in the Materials and Methods. The Mole % of the essential fatty acids (18:2, ω6 and 18:3, ω3) and the mole % of 18:2, ω6 to 18:3, ω3 is presented in the figure. B. Fig Histology of hepatosteatosis with inflammation. Liver of a mouse fed the western diet for 40 wks was fixed, embedded, sliced and stained with hematoxylin and eosin as described in the Materials and methods. The histology slide was photographed at 400x and examined and labeled by a board-certified veterinary pathologist. C. Fig Histology of hepatosteatosis: MIS & MAS. Livers were prepared from mice fed the LFD (left panel) and WD (right panel) for histology as described in Materials and Methods. Liver slices were stained with hematoxylin and eosin and photographed at 400 X, as in S1B Fig. The liver samples were examined and labeled by a board-certified veterinary pathologist. D. Fig Histology of hepatic fibrosis. Liver of a mouse fed the western diet for 40 wks was fixed, embedded, sliced and stained with Picro sirius red as described in the Materials and methods. The histology slide was photographed at 40x (left) and 100x (right) and examined and labeled by a board-certified veterinary pathologist. E. Fig Daily food consumption (A) and Cumulative calorie consumption (B). Female and male mice were fed the LFD or WD for 40 wks. Mice were weight and fed twice weekly. [A] Food consumption was quantified twice weekly and expressed as Food consumption (grams/day/mouse) for both LFD and WD fed female and male mice twice weekly. [B] Cumulative calorie consumption was calculated by multiplying the daily food consumption (grams) by the caloric density of the LFD and WD, i.e., 3 [file pone.0292432.s001.pptx]

## Slide 1
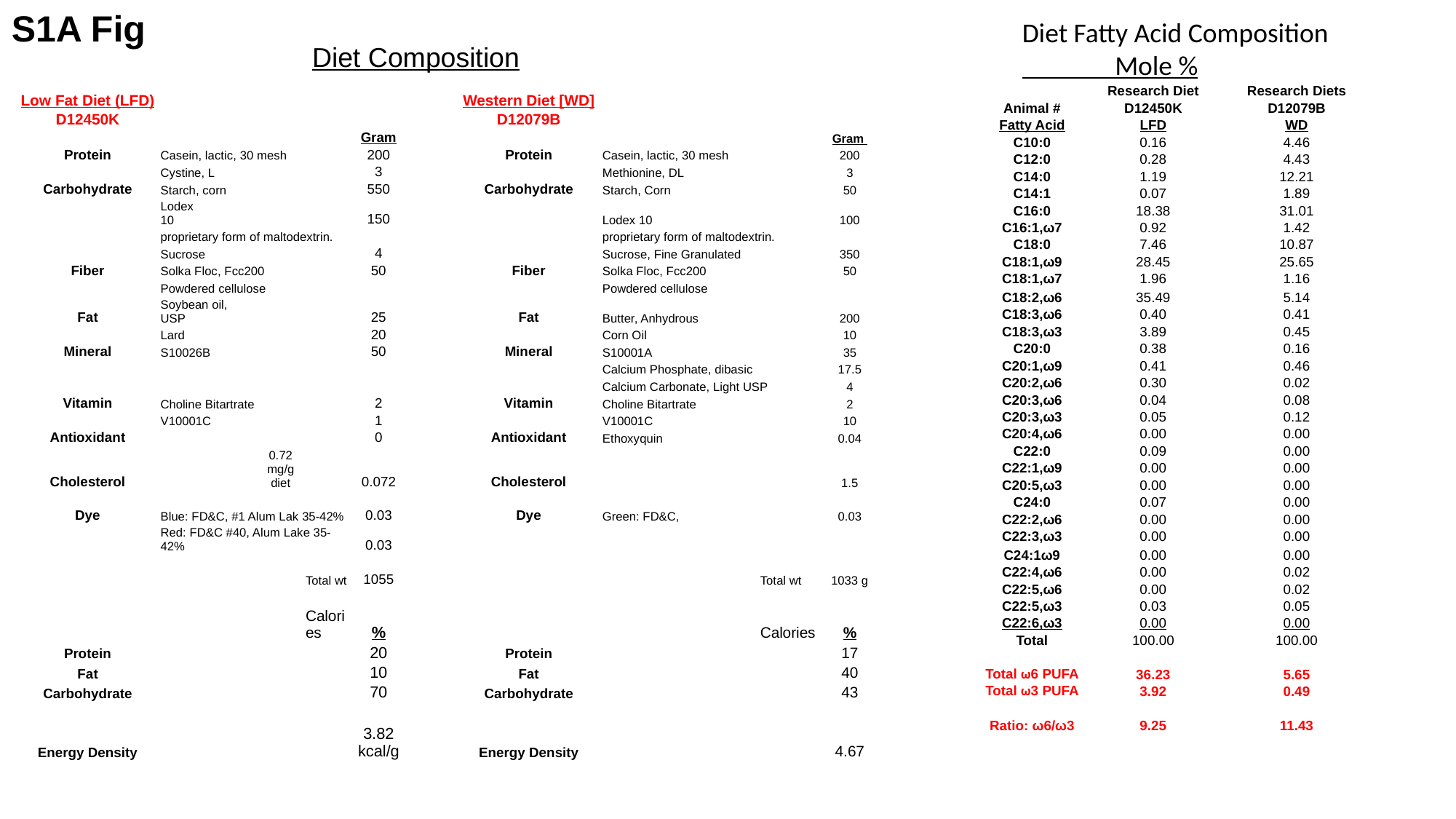

S1A Fig
Diet Fatty Acid Composition
 Mole %
Diet Composition
| | | | | | | | | | | | | | | |
| --- | --- | --- | --- | --- | --- | --- | --- | --- | --- | --- | --- | --- | --- | --- |
| Low Fat Diet (LFD) | | | | | | | | Western Diet [WD] | | | | | | |
| D12450K | | | | | | | | D12079B | | | | | | |
| | | | | | Gram | | | | | | | | | Gram |
| Protein | Casein, lactic, 30 mesh | | | | 200 | | | Protein | Casein, lactic, 30 mesh | | | | | 200 |
| | Cystine, L | | | | 3 | | | | Methionine, DL | | | | | 3 |
| Carbohydrate | Starch, corn | | | | 550 | | | Carbohydrate | Starch, Corn | | | | | 50 |
| | Lodex 10 | | | | 150 | | | | Lodex 10 | | | | | 100 |
| | proprietary form of maltodextrin. | | | | | | | | proprietary form of maltodextrin. | | | | | |
| | Sucrose | | | | 4 | | | | Sucrose, Fine Granulated | | | | | 350 |
| Fiber | Solka Floc, Fcc200 | | | | 50 | | | Fiber | Solka Floc, Fcc200 | | | | | 50 |
| | Powdered cellulose | | | | | | | | Powdered cellulose | | | | | |
| Fat | Soybean oil, USP | | | | 25 | | | Fat | Butter, Anhydrous | | | | | 200 |
| | Lard | | | | 20 | | | | Corn Oil | | | | | 10 |
| Mineral | S10026B | | | | 50 | | | Mineral | S10001A | | | | | 35 |
| | | | | | | | | | Calcium Phosphate, dibasic | | | | | 17.5 |
| | | | | | | | | | Calcium Carbonate, Light USP | | | | | 4 |
| Vitamin | Choline Bitartrate | | | | 2 | | | Vitamin | Choline Bitartrate | | | | | 2 |
| | V10001C | | | | 1 | | | | V10001C | | | | | 10 |
| Antioxidant | | | | | 0 | | | Antioxidant | Ethoxyquin | | | | | 0.04 |
| Cholesterol | | | 0.72 mg/g diet | | 0.072 | | | Cholesterol | | | | | | 1.5 |
| Dye | Blue: FD&C, #1 Alum Lak 35-42% | | | | 0.03 | | | Dye | Green: FD&C, | | | | | 0.03 |
| | Red: FD&C #40, Alum Lake 35-42% | | | | 0.03 | | | | | | | | | |
| | | | | | | | | | | | | | | |
| | | | | Total wt | 1055 | | | | | | | Total wt | Total wt | 1033 g |
| | | | | | | | | | | | | | | |
| | | | | Calories | % | | | | | | | Calories | Calories | % |
| Protein | | | | | 20 | | | Protein | | | | | | 17 |
| Fat | | | | | 10 | | | Fat | | | | | | 40 |
| Carbohydrate | | | | | 70 | | | Carbohydrate | | | | | | 43 |
| | | | | | | | | | | | | | | |
| Energy Density | | | | | 3.82 kcal/g | | | Energy Density | | | | | | 4.67 |
| | Research Diet | Research Diets |
| --- | --- | --- |
| Animal # | D12450K | D12079B |
| Fatty Acid | LFD | WD |
| C10:0 | 0.16 | 4.46 |
| C12:0 | 0.28 | 4.43 |
| C14:0 | 1.19 | 12.21 |
| C14:1 | 0.07 | 1.89 |
| C16:0 | 18.38 | 31.01 |
| C16:1,ω7 | 0.92 | 1.42 |
| C18:0 | 7.46 | 10.87 |
| C18:1,ω9 | 28.45 | 25.65 |
| C18:1,ω7 | 1.96 | 1.16 |
| C18:2,ω6 | 35.49 | 5.14 |
| C18:3,ω6 | 0.40 | 0.41 |
| C18:3,ω3 | 3.89 | 0.45 |
| C20:0 | 0.38 | 0.16 |
| C20:1,ω9 | 0.41 | 0.46 |
| C20:2,ω6 | 0.30 | 0.02 |
| C20:3,ω6 | 0.04 | 0.08 |
| C20:3,ω3 | 0.05 | 0.12 |
| C20:4,ω6 | 0.00 | 0.00 |
| C22:0 | 0.09 | 0.00 |
| C22:1,ω9 | 0.00 | 0.00 |
| C20:5,ω3 | 0.00 | 0.00 |
| C24:0 | 0.07 | 0.00 |
| C22:2,ω6 | 0.00 | 0.00 |
| C22:3,ω3 | 0.00 | 0.00 |
| C24:1ω9 | 0.00 | 0.00 |
| C22:4,ω6 | 0.00 | 0.02 |
| C22:5,ω6 | 0.00 | 0.02 |
| C22:5,ω3 | 0.03 | 0.05 |
| C22:6,ω3 | 0.00 | 0.00 |
| Total | 100.00 | 100.00 |
| | | |
| Total ω6 PUFA | 36.23 | 5.65 |
| Total ω3 PUFA | 3.92 | 0.49 |
| | | |
| Ratio: ω6/ω3 | 9.25 | 11.43 |

## Slide 2
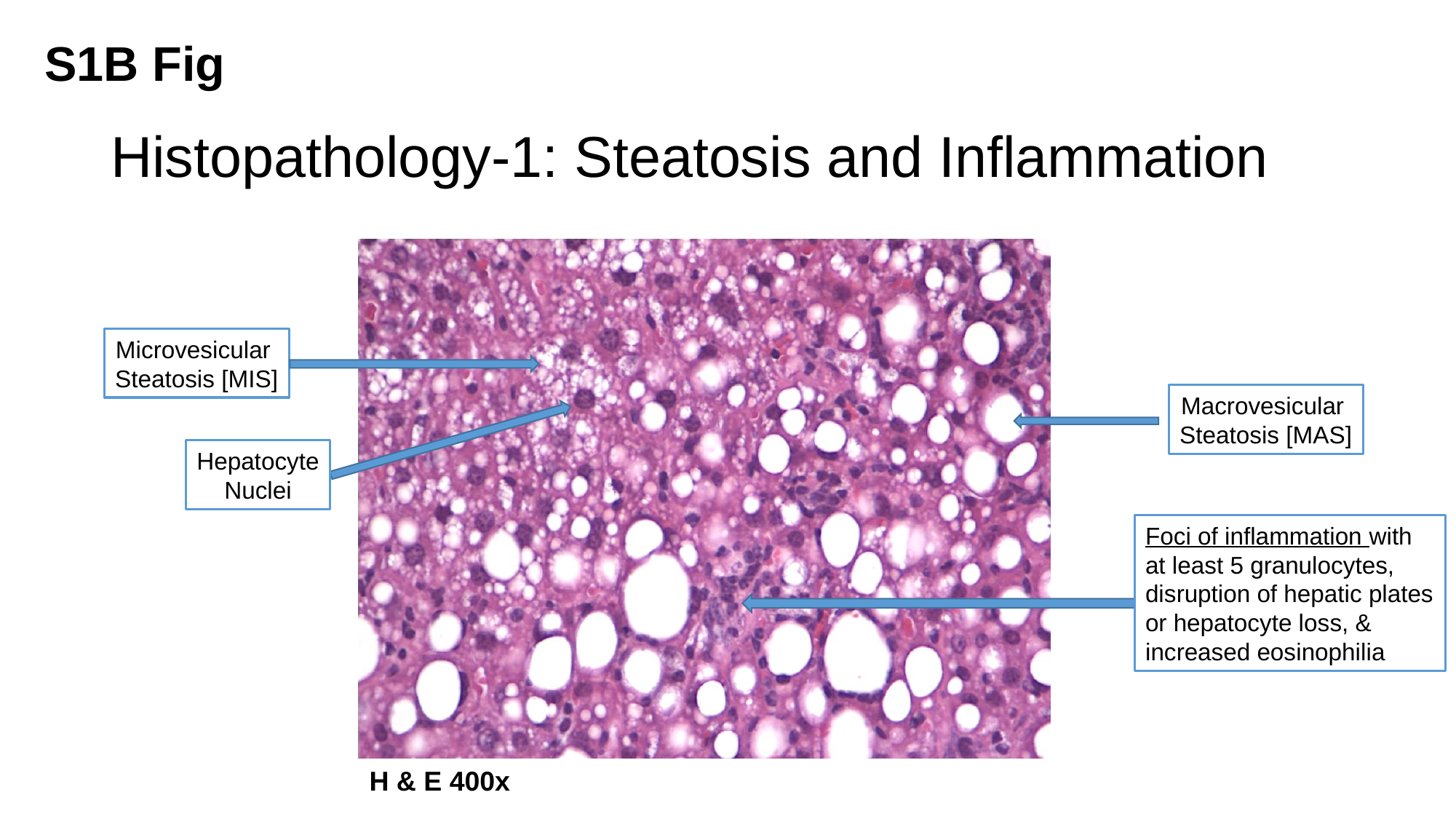

S1B Fig
# Histopathology-1: Steatosis and Inflammation
Microvesicular
Steatosis [MIS]
Macrovesicular
Steatosis [MAS]
Hepatocyte
Nuclei
Foci of inflammation with at least 5 granulocytes, disruption of hepatic plates or hepatocyte loss, & increased eosinophilia
H & E 400x

## Slide 3
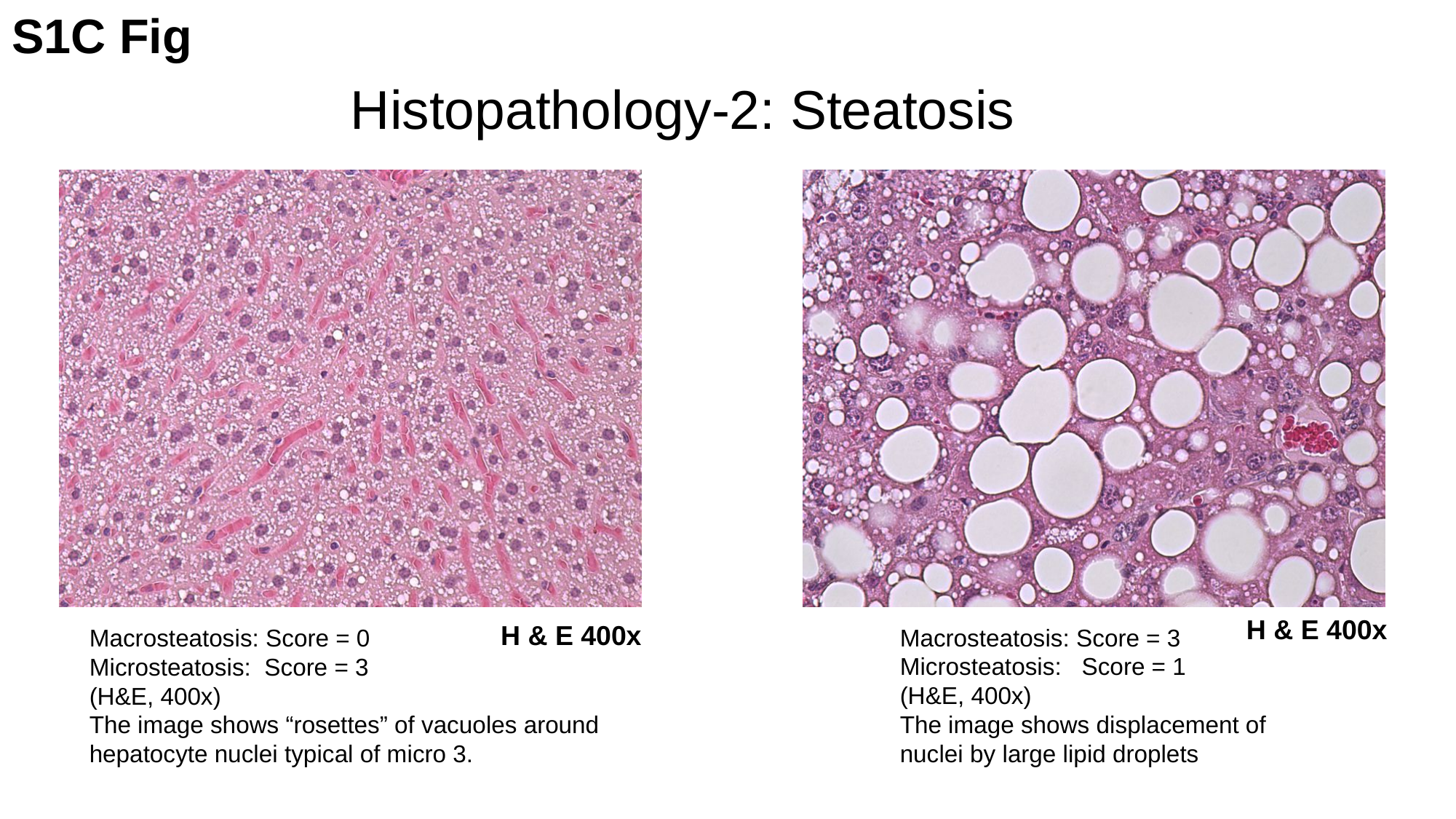

S1C Fig
Histopathology-2: Steatosis
H & E 400x
H & E 400x
Macrosteatosis: Score = 3
Microsteatosis: Score = 1
(H&E, 400x)
The image shows displacement of nuclei by large lipid droplets
Macrosteatosis: Score = 0
Microsteatosis: Score = 3
(H&E, 400x)
The image shows “rosettes” of vacuoles around hepatocyte nuclei typical of micro 3.

## Slide 4
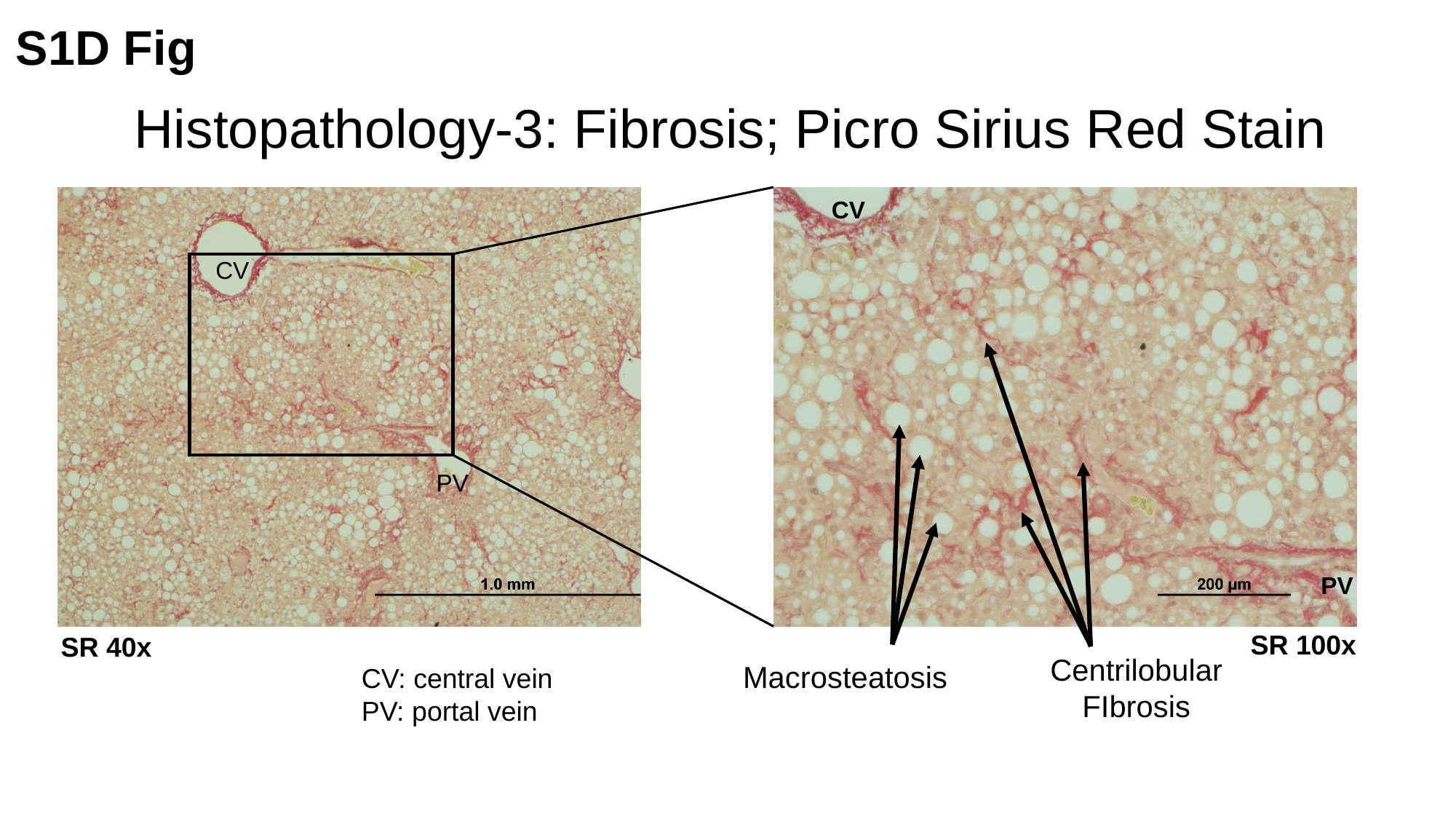

S1D Fig
Histopathology-3: Fibrosis; Picro Sirius Red Stain
CV
CV
PV
PV
SR 100x
SR 40x
Centrilobular
FIbrosis
Macrosteatosis
CV: central vein
PV: portal vein

## Slide 5
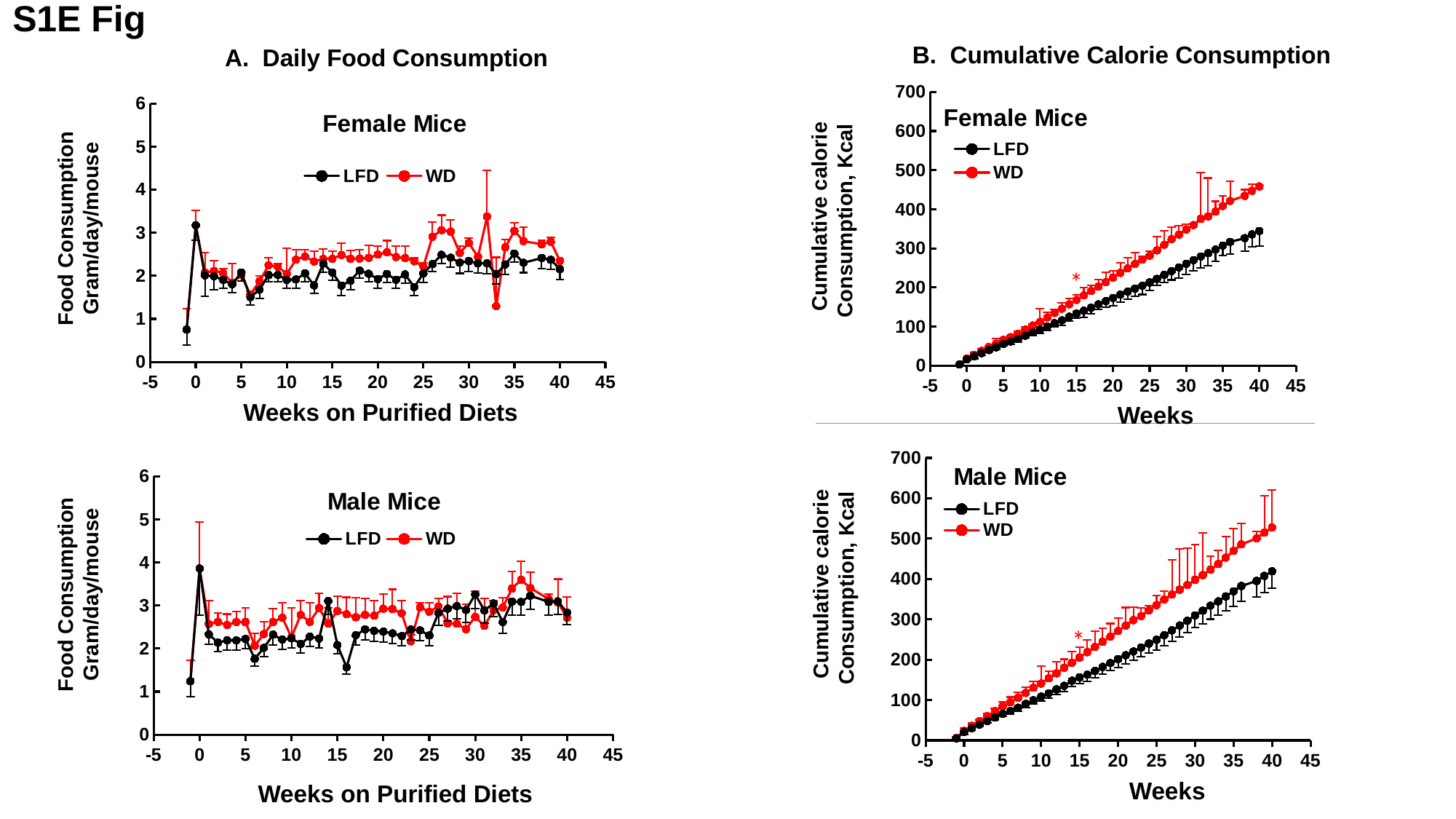

S1E Fig
 B. Cumulative Calorie Consumption
 A. Daily Food Consumption
### Chart: Female Mice
| Category | | |
|---|---|---|
### Chart: Female Mice
| Category | | |
|---|---|---| Cumulative calorie
Consumption, Kcal
Food Consumption
Gram/day/mouse
*
Weeks on Purified Diets
Weeks
### Chart: Male Mice
| Category | | |
|---|---|---|
### Chart: Male Mice
| Category | | |
|---|---|---| Cumulative calorie
Consumption, Kcal
Food Consumption
Gram/day/mouse
*
Weeks
Weeks on Purified Diets
